# Carbon dots-mediated synthesis of gold nanodendrites with extended absorption into NIR-II window for in vivo photothermal therapy

**DOI:** 10.1186/s12951-023-01887-2

**Published:** 2023-05-09

**Authors:** Guoyong Liu, Shuxian Wang, Shumin Wang, Rongrong Wu, Hui Li, Menglei Zha, Jibin Song, Yuxin Yin, Kai Li, Jing Mu, Yu Shi

**Affiliations:** 1 Department of Nuclear Medicine, Peking University Shenzhen Hospital, Shenzhen Peking University-The Hong Kong University of Science and Technology Medical Center, Shenzhen, 518036 China; 2 Department of Ultrasound, Peking University Shenzhen Hospital, Peking University, Shenzhen, 518036 China; 3 Shenzhen Key Laboratory of Smart Healthcare Engineering, Guangdong Provincial Key Laboratory of Advanced Biomaterials, Department of Biomedical Engineering, Southern University of Science and Technology, Shenzhen, 518055 China; 4 MOE Key Laboratory for Analytical Science of Food Safety and Biology, College of Chemistry, Fuzhou University, Fuzhou, 350108 China

**Keywords:** Carbon dots, Gold nanodendrites, Photothermal therapy, Photoacoustic imaging, Second near-infrared window

## Abstract

**Background:**

Photothermal therapy (PTT) in the second near-infrared (NIR-II) window has attracted extensive attention due to the benefits in high maximum permissible exposure and penetration depth. Current photothermal agents generally show a broadband absorption accompanied by a gradual attenuation of absorption in the NIR-II window, leading to poor effect of PTT. It remains a great challenge to gain photothermal agents with strong and characteristic absorption in NIR-II regions. To overcome this problem, based on carbon dots (CDs)-mediated growth strategy, we proposed a simple and feasible approach to prepare plasmonic gold nanodendrites (AuNDs) with NIR-II absorption to enhance the therapeutic effect of PTT.

**Results:**

By rationally regulating the size and branch length of AuNDs, the AuNDs exhibited a broadband absorption from 300 to 1350 nm, with two characteristic absorption peaks located at 1077 and 1265 nm. The AuNDs demonstrated desired optical photothermal conversion efficiency (38.0%), which was further applied in NIR-II photoacoustic imaging (PAI) and PTT in human colon cancer cells (HCT 116)-tumor-bearing mice model. The tumor cells could be effectively eliminated in vivo under 1064 nm laser irradiation by the guidance of PAI.

**Conclusions:**

We reported a simple but powerful synthetic method to obtain the unique AuNDs with strong and characteristic absorption peaks in the NIR-II window. This study provides a promising solution to tuning the growth of nanoparticles for bioimaging and phototherapy in the NIR-II window.

**Graphical Abstract:**

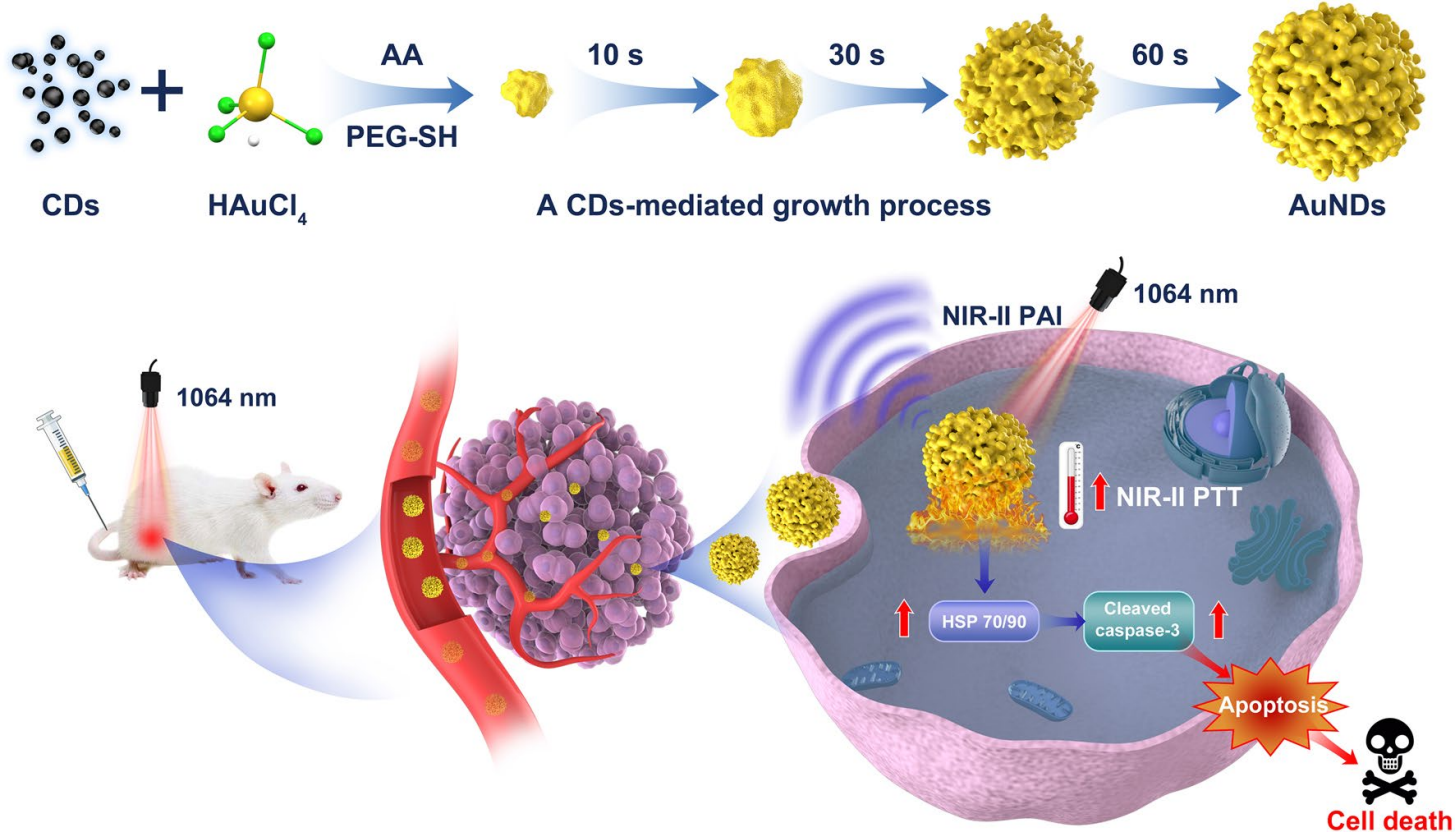

**Supplementary Information:**

The online version contains supplementary material available at 10.1186/s12951-023-01887-2.

## Introduction

Photothermal therapy (PTT) holds great potential in cancer therapy owing to the advantages of controllable treatment area, non-invasiveness, and minimal side effects [1]. During the process of PTT, light absorbers are required to convert the incoming light into heat for subsequent cancer cell ablation [2]. Compared with the first near-infrared window (NIR-I, 700–900 nm), the light in the second near-infrared window (NIR-II, 1000–1700 nm) has the virtues of higher maximum permissible exposure (MPE), better signal-­to-­background ratio, lower tissue scattering and higher penetration depth [3]. Currently, there is an increasing interest to develop various types of photothermal agents, like metal chalcogenides, two-dimensional materials, semiconducting polymers, etc. that absorb NIR-II light for deep-tissue photoacoustic imaging (PAI) and PTT [4, 5].

As a noble metal material, gold nanoparticles play an important role in bioimaging, sensing and cancer treatment [6]. Due to the adjustable morphologies and localized surface plasmon resonance (LSPR) effect, gold nanomaterials display unique optical properties [7]. Gold nanostructures with various morphologies have been synthesized, such as spheres, rods, stars, rings, dendrites, cages, and so on [8]. However, most gold nanostructures with characteristic absorption peak mainly located in the NIR-I window (less than 1000 nm) [9, 10], and the bioapplications for NIR-II phototherapy are quite limited [11]. Tremendous efforts have been made in tuning the absorption from NIR-I to NIR-II by modulating the morphology of gold-based nanomaterials [12]. For example, by tuning the gap between the Au nanorod and the Au/Ag nanoshell, Yeh et al. prepared a rod-in-shell gold structure with two NIR SPR bands beyond 1000 nm [13]. However, the laser power density (3 W cm^−2^) is not suitable for the in vivo applications since the larger MPE for skin exposure is 1 W cm^−2^ at 1064 nm (American National Standard for Safe Use of Lasers, ANSI Z136.1-2007) [14, 15]. Therefore, it is necessary to develop a new synthetic approach to regulate the absorption of gold nanostructures in NIR-II for PTT.

Carbon dots (CDs) are one type of emerging zero-dimensional carbon-based nanoparticles with widespread applications in imaging, sensing, theranostics, energy storage and catalysis [16–18]. CDs have attracted extensive attention due to their excellent optical properties, low toxicity, good biocompatibility and high chemical stability [19]. More importantly, the abundant functional groups on the surface of CDs, such as –NH_2_, –COOH and –OH, play a crucial role in regulating the morphology of nanomaterials by involving in the nucleation and growth processes of metal compounds [20]. By controlling the ratio of CDs/NiCo_2_O_4_ composites in the hydrothermal process, Xiong et al. fabricated a series of CDs/NiCo_2_O_4_ composites with different morphologies, including sea urchins-like, chestnut-like, flower-like and bayberry-like [21]. Using CDs as the structural regulator of metal oxides (MOs), porous pomegranate-like MOs/CDs composites could be prepared based on CDs-induced in situ growth mechanism [22]. Given the great potential of CDs in regulating the morphology of nanomaterials, investigations on new methods to tune the absorbance of gold-based nanomaterials are highly desired.

In this work, we provide a simple but powerful approach to prepare gold nanodendrites (AuNDs) with strong and characteristic NIR-II absorption beyond 1000 nm based on CDs-mediated growth strategy. By changing the amount of auxiliary material CDs, reducing agent ascorbic acid and stabilizer poly(ethylene glycol)methyl ether thiol (PEG-SH), the AuNDs displayed unique multi-branched morphology and ideal NIR-II absorption peak at 1077 nm and 1265 nm. The underlying growth mechanism was systematically investigated, and the relationship between the morphology of AuNDs and the NIR-II absorption was further confirmed by theoretical calculations. The prepared AuNDs demonstrated good optical photothermal conversion efficiency, which was further applied in NIR-II PAI and PTT in human colon cancer cells (HCT 116)-tumor-bearing mice model. Overall, this work not only developed a CDs-mediated synthetic method to obtain AuNDs with extended absorption into NIR-II window, but also explored their potential applications in NIR-II PAI and PTT in vivo.

## Materials and methods

### Chemicals and materials

HAuCl_4_·3H_2_O, phosphate buffered saline (PBS), ascorbic acid, glutaraldehyde, osmic acid, ethylenediamine (EDA) and 4-aminophenol (AP) were supplied by Aladdin Industrial Corporation (China). Poly(ethylene glycol)methyl ether thiol (PEG-SH, 2 k) was obtained from Ponsure Biotechnology (China). Cell counting kit-8 (CCK-8), calcein acetoxymethyl ester/propidium iodide (calcein AM/PI) staining kit, polyvinylidene fluoride membranes and RIPA lysis buffer were purchased from Beyotime Biotechnology (China). Dulbecco’s modified Eagle’s medium (DMEM), penicillin–streptomycin and fetal bovine serum (FBS) were acquired from Corning (USA). Anti-caspase-3 antibody, anti-heat shock protein (HSP) 70 antibody, anti-HSP90 antibody, anti-β-tubulin antibody and horseradish peroxidase (HRP)-labeled secondary antibody were obtained from abcam Corporation (UK). Bovine serum albumin (BSA) was purchased from sigma-aldrich Corporation (USA). All chemicals were used as received. Milli-Q purified water (18.2 MΩ cm) was used in all aqueous solutions.

### Apparatus and characterizations

UV–vis-NIR spectra were carried out on a Shimadzu UV-2600 spectrometer (Japan). Transmission electron microscopy (TEM) images were acquired on a Tecnai G2 F20 instrument (FEI, USA). X-ray photoelectron spectroscopy (XPS) measurements and X-ray diffraction (XRD) pattern were obtained on Thermo ESCALAB VG Scientific 250 (UK) and Bruker D8 ADVANCE (Germany), respectively. Fourier transform infrared spectroscopy (FT-IR) measurements were conducted on a Bruker Optics VERTEX 70 spectrometer (Germany). The dynamic light scattering (DLS) measurements were acquired on a Malvern Zetasizer Nano ZS90 instrument (UK). The percentages of apoptotic cells were determined on a flow cytometry (Beckman Coulter, USA). The concentrations of gold ions were determined by inductively coupled plasma mass spectrometry (ICP-MS) on an Agilent 7700 series equipment (USA). Thermal images were acquired by a FLIR E6 thermal camera. Photoacoustic imaging (PAI) was carried out on a Vevo LAZR-X multi-mode ultrasound/PAI system (VisualSonics, Canada).

### Synthesis of AuNDs

2 mL of 4-aminophenol aqueous solution (0.01 M) was added to 100 mL of ethylenediamine aqueous solution (0.01 M), followed by keeping 2 h at room temperature to obtain CDs. The resultant solution was adjusted to neutral solution by 150 μL of HCl (6 M). Next, 1 mL of 20 mg/mL HAuCl_4_ aqueous solution and 1 mL of ascorbic acid aqueous solution (1 M) were sequentially added to the neutral solution. After 1 min, 1 mL of 50 mg/mL PEG-SH aqueous solution was added into the mixture solution and stirred at room temperature for 2 h. The AuNDs were acquired by centrifugation and washing.

### Electromagnetic simulations

Finite-Difference Time-Domain (FDTD) software was utilized to calculate the absorption spectrum and electric field distribution of AuNDs. In the calculation process, the Johnson and Christy model in the material library was selected as the dielectric permittivity of gold. The perfect matching layer condition was applied in the x, y, and z directions. In addition, 1 nm × 1 nm × 1 nm mesh was used to ensure accurate calculation results. Total-field scattered-field plane wave was chosen as excitation source.

### Photothermal performance of AuNDs under 1064 nm laser

To measure the photothermal performance of AuNDs under 1064 nm laser, 200 μL of AuNDs aqueous suspensions (10, 30, 50, 70, 100 and 150 μg mL^−1^) were respectively placed in the 250 μL centrifuge tubes and irradiated by 1064 nm laser (1 W cm^−2^) for 6 min. The digital photothermal imaging system was used to visually monitor the temperature changes of AuNDs aqueous suspensions. Heating of AuNDs aqueous suspension for 6 min and then naturally cooling it through four repeated lasers on/off cycles were carried out to evaluate its photothermal stability. The concentrations of AuNDs aqueous suspensions used to measure photothermal stability were 100 μg mL^−1^ for 1064 nm laser (1 W cm^−2^). The determination of photothermal conversion efficiency (*η*) was referring to the previous report [23].

### In vitro tissue penetration

Chicken breast tissue with different thickness (0, 2, 4, 6, 8 and 10 mm) were utilized as model biological tissues. 200 μL of AuNDs aqueous solution (150 μg mL^−1^) was covered with different tissue thickness, and then irradiated with an 808 nm or 1064 nm laser (1 W cm^−2^) for 6 min from the top. The temperature was monitored by a thermal imaging camera.

### Cell uptake of AuNDs

HCT 116 cells (1 × 10^6^ cells/well) were seeded in 6-well plates and cultured in DMEM containing 10% FBS and 1% penicillin–streptomycin at 37 °C under 5% CO_2_ for 12 h. Then, the cells were treated with fresh medium containing 100 μg mL^−1^ AuNDs for 4 h. After that, the cells were collected and fixed with 2.5% glutaraldehyde solution overnight. Subsequently, the cell pellets were washed with PBS, and then fixed with 1% osmic acid solution for 1 h. After washing, the cell pellets were dehydrated with a series of graded ethanol. Afterward, the cell pellets were embedded in epoxy for 24 h, and then cut into thin section (70 nm) for bio-TEM observation.

### Cytotoxicity and photothermal killing effect of AuNDs

HCT 116 cells (5 × 10^3^ cells/well) were seeded in 96-well plates and cultured for 24 h. AuNDs with various concentrations from 0 to 100 μg mL^−1^ were added into the wells and then cultured for 24 h. The well was irradiated with/without 1064 nm laser (1.0 W cm^−2^) for 6 min. Subsequently, the media were replaced with fresh culture media and incubated for another 24 h. Then, 10 µL CCK-8 solution was added to each well and incubated for 2 h. The cell viability was determined by microplate reader at the wavelength of 450 nm.

### Live/dead staining and flow cytometry assay

HCT 116 cells (5 × 10^3^ cells/well for fluorescence imaging or 1 × 10^6^ cells/well for flow cytometry analysis) were incubated in 96-well or 6-well plates for 24 h. The cells were divided into 4 groups: (1) PBS, (2) 1064 nm laser irradiation, (3) AuNDs and (4) AuNDs + 1064 nm laser irradiation. Group (3) and group (4) were added fresh culture media containing 100 μg mL^−1^ AuNDs, and then cultured for 4 h. Subsequently, group (2) and group (4) were treated with 1064 nm laser irradiation (1.0 W cm^−2^, 6 min), respectively. After 24 h, the media were replaced with calcein-AM (2 μM)/PI (4.5 μM) solution or Annexin V-FITC (5 μL)/PI (10 μL) solution. After further incubation for 15 min, the cells were detected by fluorescence microscope or flow cytometry.

### Western blot analysis

The expressions of HSP70, HSP90, cleaved caspase-3 and β-tubulin were detected by western blot. HCT 116 cells were seeded in 6-well plates for 24 h. Then, the cells were treated with various conditions: (1) PBS, (2) 1064 nm laser irradiation (1.0 W cm^−2^, 6 min), (3) AuNDs (100 μg mL^−1^) and (4) AuNDs (100 μg mL^−1^) + 1064 nm laser irradiation (1.0 W cm^−2^, 6 min). After incubated for another 24 h, the cells were collected and then lysed in RIPA lysis buffer to acquire protein. The protein concentration was determined by BCA protein assay kit (abcam, UK). 30 µg of protein was loaded onto 10% or 15% sodium dodecyl sulfate polyacrylamide gel electrophoresis and transferred to PVDF membranes. After blocked with BSA for 2 h, the membranes were incubated with the primary antibodies at 4 °C overnight, and then treated with HRP-labeled secondary antibody for 2 h. Finally, images were obtained on a chemiluminescence gel imaging system (ChemiDoc XRS +, Bio-Rad, USA).

### Photoacoustic measurements of AuNDs in vitro and in vivo

AuNDs aqueous solutions with different concentrations (0, 0.25, 1, 2, 4 and 5 mg/mL) were placed in a photoacoustic system to measure the in vitro photoacoustic signal intensity. For PAI in vivo, 400 μL AuNDs (3 mg/mL) were injected into HCT 116 tumor-bearing female mice through the tail vein to monitor the photoacoustic signal in the tumor region at different time points (0, 4, 8, 12, 24 and 48 h). The above photoacoustic signals in vitro and in vivo were acquired at the wavelength of 1250 nm.

### In vivo anti-tumor assessment

To establish the HCT 116 colon tumor-bearing mouse model, female BALB/c nude mice (4 ~ 5 weeks) were subcutaneously inoculated with 2 × 10^6^ HCT 116 cells into the right flank. When the tumor volume reached ~ 70 mm^3^, the mice were randomly divided into 4 groups (n = 5): (1) i.v. injected with 400 μL of PBS, (2) i.v. injected with 400 μL of PBS and then irradiated by 1064 nm laser at 12 h post injection, (3) i.v. injected with 400 μL of Au NDs (3 mg/mL), 4) i.v. injected with 400 μL of Au NDs (3 mg/mL) and then irradiated by 1064 nm laser at 12 h post injection. The nanomaterials were injected into HCT 116 tumor-bearing mice through the tail vein once. The 1064 nm laser power density was 1 W cm^−2^ for 15 min. The temperature of tumors was monitored by a FLIR E6 thermal camera. The tumor sizes and body weights were then determined every other day. On day 14 after treatment, the tumor tissues were dissected and recorded.

### Ex vivo histology assessment

The mice were killed and tumor tissues were obtained for slicing at 72 h after different treatments. Then tumor slices were stained with hematoxylin and eosin (H&E) and terminal deoxynucleotidyl transferase deoxyuridine triphosphate (dUTP) nick end labeling (TUNEL). The H&E- and TUNEL-stained images were observed by using optical microscope.

### Hemolysis assay

The red blood cells (RBCs) were separated from the whole blood of Balb/c mice and then washed several times with PBS. The diluted cell suspension (20% in PBS) was mixed with AuNDs at different concentrations. The deionized water and PBS were utilized as positive and negative controls, respectively. All samples were maintained at 37 °C for 3 h. Then, the mixtures were centrifuged and the supernatants collected for microplate reader measurement at 540 nm. The percentage of hemolysis rate was calculated as follows: Hemolysis rate (%) = (Asample − Anegative control)/(Apositive control − Anegative control) × 100%

### In vivo toxicity

Healthy BALB/c mice (6 mice in total) were intravenously injected with AuNDs (400 μL, 3 mg/mL) or PBS (400 μL) as control. All mice were sacrificed on day 14 after administration. The blood samples were collected from eyelids for blood routine and blood biochemistry, and the major organs were excised for histomorphology analysis by H&E.

## Results and discussion

### Synthesis and characterization of AuNDs

**Fig. 1:**
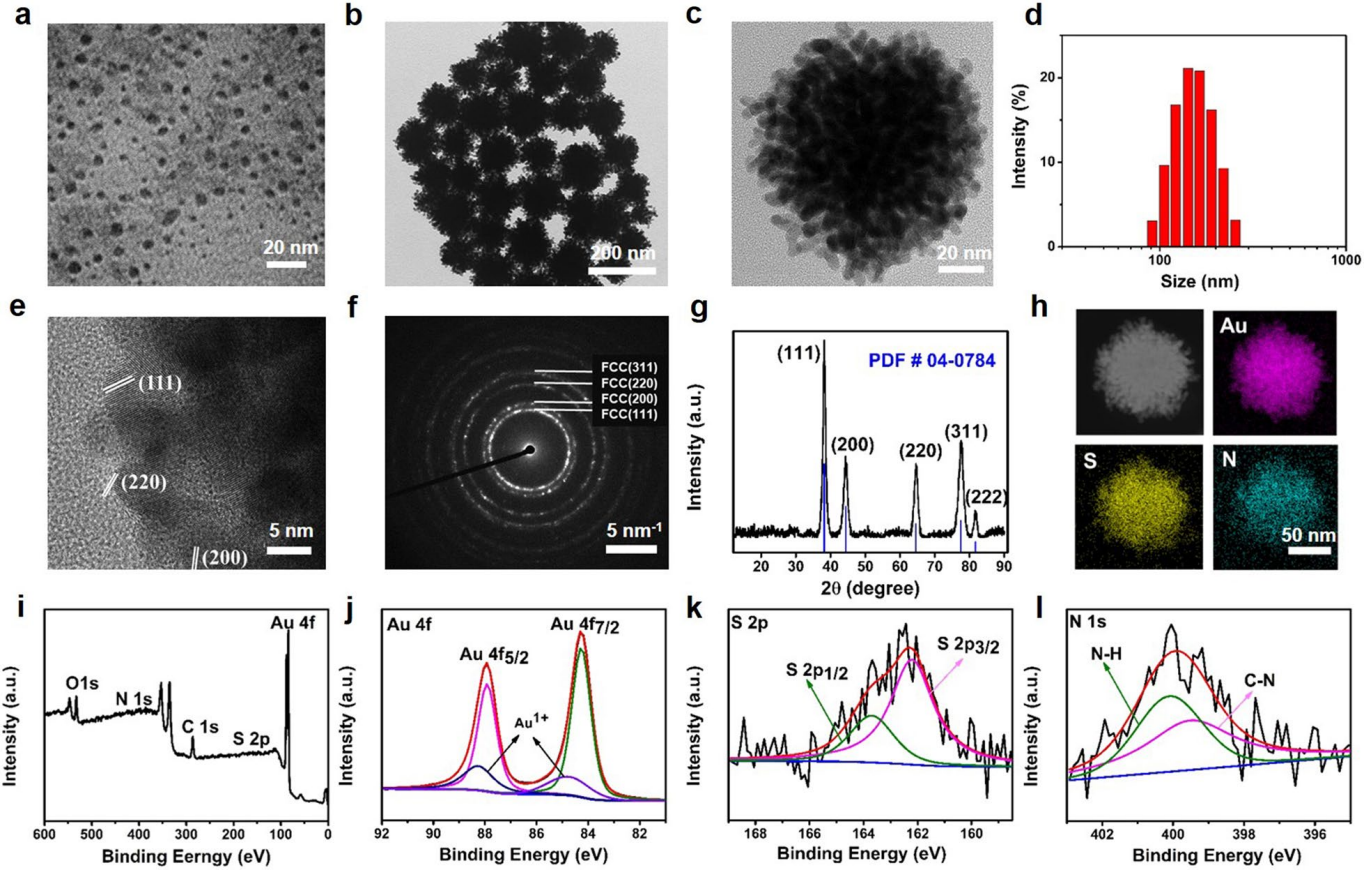
**a** TEM image of CDs. **b-l** Characterization of AuNDs. **b**-**c** TEM images. **d** DLS measurements. **e** High-resolution TEM image. **f** Selected area electron diffraction pattern. **g** XRD pattern. **h** Dark-field image and elemental mappings of AuNDs, including Au, S, and N elements. **i** XPS spectra of full-spectrum, **j** Au 4f, **k** S 2p, and **l** N 1 s

In this study, a CDs-mediated approach was utilized to synthesize the AuNDs. First of all, the CDs were prepared by simply mixing the solutions of ethylenediamine (EDA) and 4-aminophenol (AP) at room temperature for 2 h. Then, transmission electron microscopy (TEM), Fourier-transform infrared spectroscopy (FT-IR) and X-ray photoelectron spectroscopy (XPS) of CDs were carried out to investigate the size, morphology, and chemical composition. As shown in Fig. 1a, CDs displayed uniform nanoparticles with an average size of 5.7 nm. Functional groups, such as C-O (1230 cm^−1^), C-N (1342 cm^−1^), C=C (1514 cm^−1^), C=O/C=N (1661 cm^−1^), O–H/N–H (3321 cm^−1^) were found in FT-IR spectrum (Additional file 1: Fig. S1) [24]. XPS results revealed that CDs mainly contain C (285.1 eV), N (400.1 eV) and O (532.1 eV) three elements (Additional file 1: Fig. S2a) [25]. Additionally, N 1 s spectra of CDs exhibited C=N (398.3 eV), N–H (399.5 eV) and C–N (400.4 eV) bonds [26], validating Schiff base reaction between AP and EDA (Additional file 1: Fig. S2b) [27]. All above results clearly demonstrated successful preparation of CDs.

After the synthesis of CDs, HAuCl_4_ and the reducing agent ascorbic acid were added sequentially at pH of 7, followed by the addition of PEG-SH to increase the stability of AuNDs in aqueous solutions. TEM and dynamic light scattering (DLS) measurements revealed that the AuNDs exhibited branched dendritic nanocluster structure with an average hydrodynamic diameter of 155 nm (Fig. 1b–d). Then high-resolution TEM image implied an obvious lattice structure with the lattice fringes of 0.235, 0.204, and 0.144 nm, which corresponded to the *d*-spacing of the (111), (200), and (220) plane of Au, respectively (Fig. 1e) [28]. The concentric rings of spots in the selected-area electron diffraction (SAED) pattern represented the face-centered cubic (FCC) crystal structure of Au (Fig. 1f) [29]. To further verify the crystal structure of AuNDs, X-ray power diffraction (XRD) measurements were performed and the result was in accordance with the crystal phases of Au (JCPDS: 04-0784) (Fig. 1g) [30]. Energy-dispersive X-ray spectroscopy (EDS) and XPS measurements further confirmed the distribution of C (286.8 eV), N (400.0 eV), O (533.2 eV), Au (84.3 and 87.9 eV) and S (162.4 eV) elements (Fig. 1h, i and Additional file 1: Fig. S3). The two peaks at 87.9 and 84.3 eV in Au 4f spectrum demonstrated the existence of metallic Au^0^ 4f_5/2_ and Au^0^ 4f_7/2_ bands, respectively (Fig. 1j) [31]. In addition, two peaks at 88.3 and 84.8 eV in Au 4f spectrum belong to Au^1+^ 4f_5/2_ and Au^1+^ 4f_7/2_, respectively [32]. In the S 2p spectrum, the peaks at 162.1 (S 2p_3/2_) and 163.6 eV (S 2p_1/2_) belong to sulfide (S^2−^) and metal-deficient sulfide, respectively (Fig. 1k) [33]. The N 1 s spectrum implied the existence of C-N (399.5 eV) and N–H (400.0 eV) species, which could be likely attributed to EDA (Fig. 1l).

To obtain ideal AuNDs with optimal size and absorption properties, various experimental parameters, including concentrations of CDs (controlled by the concentration of AP), HAuCl_4_, HCl, and PEG-SH, were tuned respectively and characterized by absorption spectra and TEM images. The optimum conditions of AP, HAuCl_4_, HCl, and PEG-SH were 200 µM, 1 mL, 150 µL, and 50 mg/mL, respectively (Additional file 1: Fig. S4–S7). It should be noted that AuNDs cannot be successfully synthesized in the absence of AP. In addition, the increase of CDs amount could promote the formation of branches of AuNDs (Additional file 1: Fig. S8). These results clearly indicated that the addition of CDs is necessary for the formation of nanodendrite morphology. We also found that the concentration of AA was critical in tuning the absorption spectra, size and morphology of AuNDs. In the absence of AA, spherical gold nanoparticles with a size of 87 nm were obtained (Additional file 1: Fig. S9a). As the concentration of AA increased from 10 to 200 mM, the average sizes of AuNDs changed from 253 to 109 nm. Meanwhile, the branches of AuNDs gradually became longer and characteristic absorption peaks at 1077 nm and 1265 nm occurred (Additional file 1: Fig. S9b–f and S10).

**Fig. 2:**
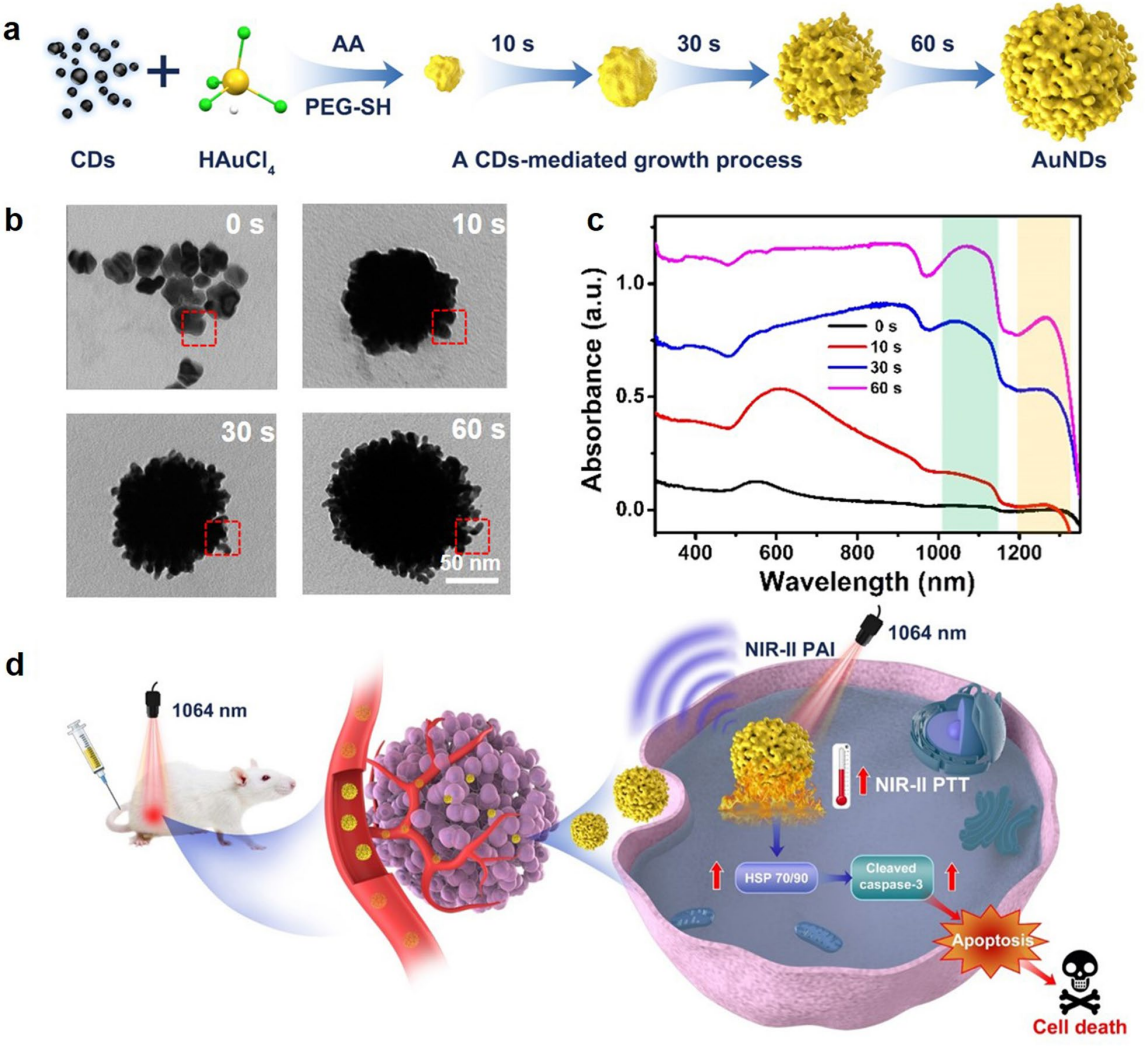
**a** Schematic illustration of synthetic process of AuNDs. TEM images **b** and absorption spectra **c** of AuNDs growth process within 1 min. **d** Illustration of AuNDs applied in NIR-II PAI-guided PTT in vivo

What’s more, the time course of AuNDs formation was investigated in detail. As depicted in Fig. 2a, b, AuNDs were small and irregular nanoparticles at the beginning of the reaction. After 10 s, the gold nanoparticles gradually became larger and obvious dendritic structure appeared at 30 s. AuNDs with an average size of around 120 nm and longer branches were formed at 60 s. The absorbance at 1077 and 1265 nm gradually enhanced with increasing reaction time (Fig. 2c). According to the above results, we speculated the synthetic mechanism of AuNDs as follows: At the beginning, CDs provided attachment sites for Au^3+^, and AA reduced Au^3+^ to metallic Au. With the extension of reaction time, metallic Au continuously fused to form large nanoparticles. However, due to the existence of CDs, misorientations occurred at the interface of gold nanoparticles, resulting in lattice mismatches and crystal defects, thus forming a multi-branched structure [34]. Surprisingly, such unique structure enabled strong absorption with two characteristic peaks in the NIR-II window, which allowed for promising applications in deep-tissue PA and PTT (Fig. 2d).

### Photothermal properties of AuNDs

**Fig. 3:**
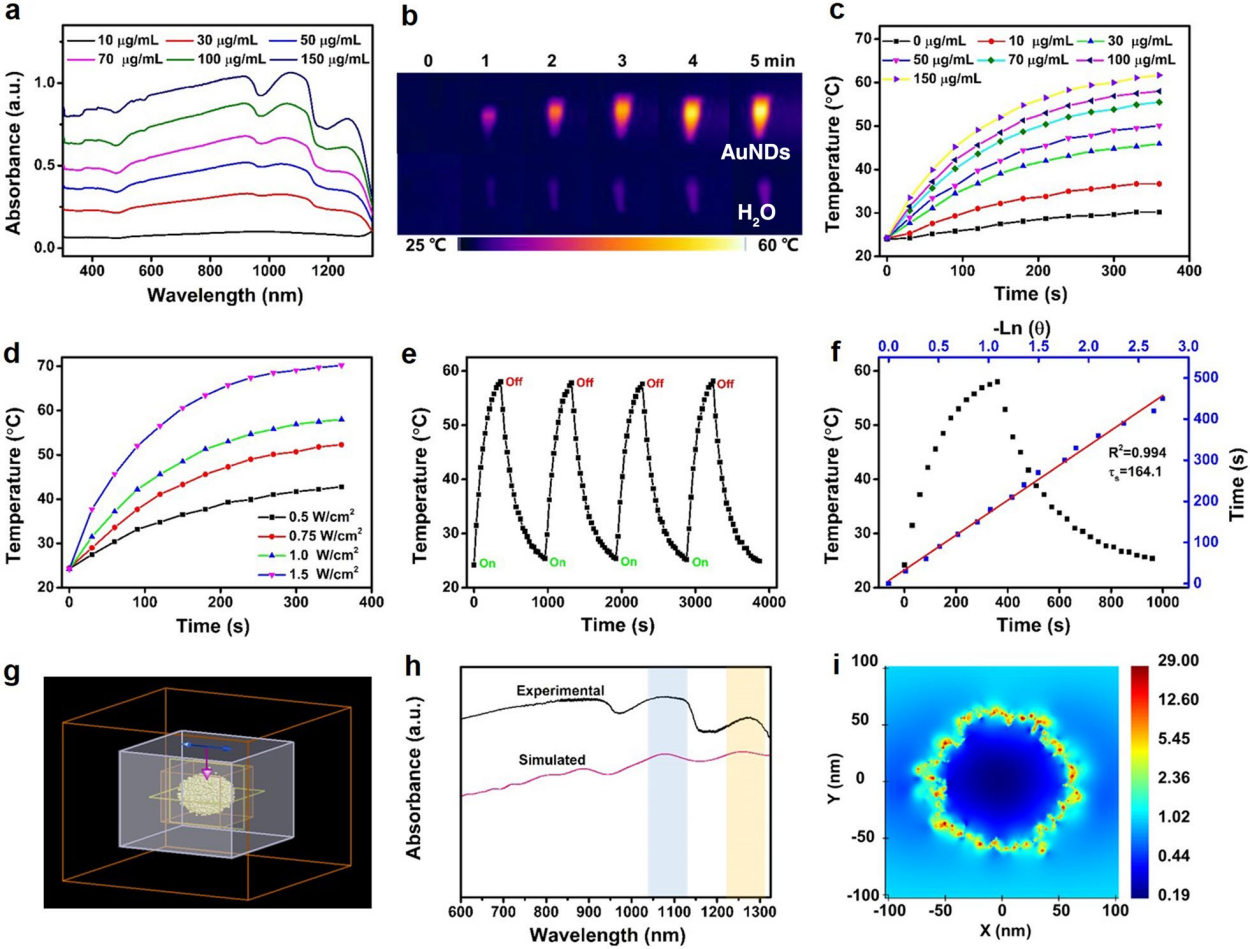
Photothermal performance of AuNDs. **a** UV–vis–NIR absorption spectra of different concentrations of AuNDs aqueous solution. **b** Infrared thermal images of AuNDs aqueous solution (100 µg mL^−1^) and H_2_O under 1064 nm laser irradiation (1.0 W cm^−2^) for 5 min. **c** Temperature elevation curves of different concentrations of AuNDs aqueous solution under 1064 nm laser irradiation (1.0 W cm^−2^) for 6 min. **d** Heating curves of AuNDs aqueous solution (100 µg mL^−1^) under 1064 nm laser irradiation at different power densities. **e** Photothermal stability of AuNDs aqueous solutions under 1064 nm laser through four cycles of heating and cooling processes. **f** Black: Temperature profile of the AuNDs solutions irradiated by 1064 nm laser (1.0 W cm^−2^) for 6 min, followed by natural cooling. Blue: The linear fitting of time versus –ln (θ) from the cooling period. **g** Model of AuNDs. **h** Experimental and simulated absorption spectra of AuNDs. **i** Electric near-field enhancement distribution for AuNDs at 1064 nm

To investigate the photothermal property of AuNDs, optical absorption measurements were first performed. As shown in Fig. 3a, the AuNDs aqueous solution exhibited a broadband absorption from 300 to 1350 nm, with two characteristic absorption peaks located at 1077 and 1265 nm. In addition, there was a positive correlation between the absorption intensities at 1064 nm and concentrations of AuNDs (Additional file 1: Fig. S11). Then obvious temperature increase was observed under 1064 nm laser irradiation (1.0 W cm^−2^) for 5 min (Fig. 3b). The photothermal-heating curves exhibited both concentration and laser power-dependent pattern, and the temperature of AuNDs aqueous solution (150 µg mL^−1^) could rapidly increase from 24 to 61.7 °C within 6 min (Fig. 3c, d). Through 4-cycle repeated heating and cooling measurements, the maximum steady-state temperature remained unchanged (Fig. 3e). Meanwhile, the absorption spectra of AuNDs exhibited no significant changes before and after exposure to 1064 nm laser for 10 min (Additional file 1: Fig S12), implying good photothermal stability of AuNDs.

Meanwhile, the photothermal conversion efficiency (*η*) of the AuNDs was calculated to be 38.0% (Fig. 3f), which was comparable to the previous inorganic photothermal agents (Additional file 1: Table S1). To signify the superiority of NIR-II light over NIR-I light in tissue penetration, the deep-tissue photothermal experiment of AuNDs was carried out, as depicted in Additional file 1: Fig. S13a. With the increase of chicken breast tissue thickness, the temperature increment of AuNDs irradiated by 1064 nm laser or 808 nm laser decreased (Additional file 1: Fig. S13b). However, at the same thickness, the temperature increment caused by 1064 nm laser was higher than that of 808 nm laser, implying the stronger tissue penetration ability of NIR-II light than that of NIR-I light [35]. In order to better understand the photothermal source of AuNDs, the absorption spectrum and electric field distribution of AuNDs were calculated by Finite-Difference Time-Domain (FDTD) software [36]. The simulated spectrum was consistent with the experimental spectrum (Fig. 3g, h), especially for two characteristic absorption peaks at 1077 and 1265 nm in NIR-II window, suggesting the reliability of this model. As shown in Fig. 3i, the electric field results confirmed the existence of hot spots, which was likely attributed to the abundant plasmon coupling occurred between the closely spaced branches [37, 38]. Such enhancement of electric field resulted in strong NIR-II absorption and localized plasmon heating, which could be considered as a good candidate for NIR-II photoacoustic and photothermal agents.

**Fig. 4:**
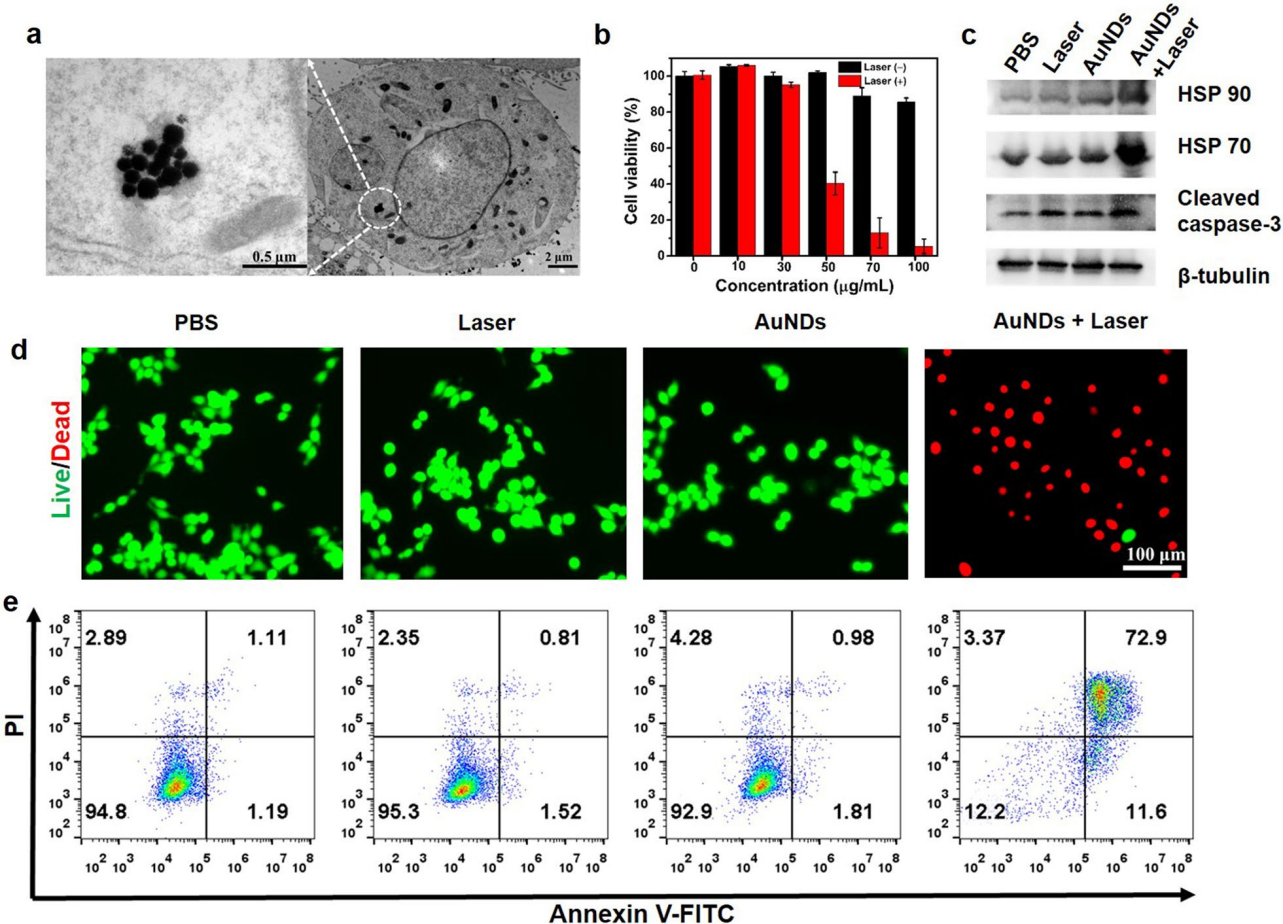
**a** The bio-TEM images of AuNDs in intracellular localization after 4 h incubation, showing cellular uptake of AuNDs. **b** Cell viability of HCT 116 cells after treatment by different concentrations of AuNDs with or without NIR-II irradiation (1064 nm, 6 min). **c** Western blot analysis, **d** Live/dead staining and **e** flow cytometry analysis of HCT 116 cells after different treatments, including PBS, 1064 nm laser irradiation, AuNDs, and AuNDs under 1064 nm laser irradiation

After verifying the photothermal effect of AuNDs, we next investigated their intracellular behaviors in HCT 116 cells. The intracellular uptake of AuNDs was evaluated by bio-TEM after incubation with HCT 116 cells for 4 h. As shown in Fig. 4a, the AuNDs could be efficiently internalized into HCT 116 cell [39], and the AuNDs were relatively stable in complex cellular environment (Additional file 1: Fig. S14). To further testify the therapeutic effect, the cell viability was determined by a standard cell counting kit-8 (CCK-8) assay. As shown in Fig. 4b, no obvious cytotoxicity of AuNDs was observed, even at a high concentration of 100 μg/mL, suggesting their good biocompatibility. Upon exposure to 1064 nm laser irradiation, tumor cell proliferation was significantly inhibited in a dose-dependent manner and the cell viability was decreased to only 5.3% at the concentration of 100 μg/mL, clearly indicating the good photothermal cytotoxicity of AuNDs (Fig. 4b). To gain insight into the cellular response during PTT, relevant protein expression was assessed by Western blot. As shown in Fig. 4c, heat shock protein (HSP) 70, HSP90 and cleaved caspase-3 showed increased expression levels in cells treated with AuNDs + laser irradiation, implying the activation of apoptotic pathway during the process [40, 41]. Besides, cell death mechanism was further investigated by both live/dead cell staining and apoptosis staining. Apparently, strong red signals under the fluorescence microscope in the AuNDs + laser treated group demonstrated remarkable photothermal killing effect of AuNDs (Fig. 4d) [42]. Quantitative flow cytometry assay by using the annexin V-FITC/PI apoptosis detection kit further revealed that AuNDs-mediated hyperthermia mainly induced the late apoptosis or necrosis processes (Fig. 4e) [43].

### In vitro and in vivo PA imaging

**Fig. 5:**
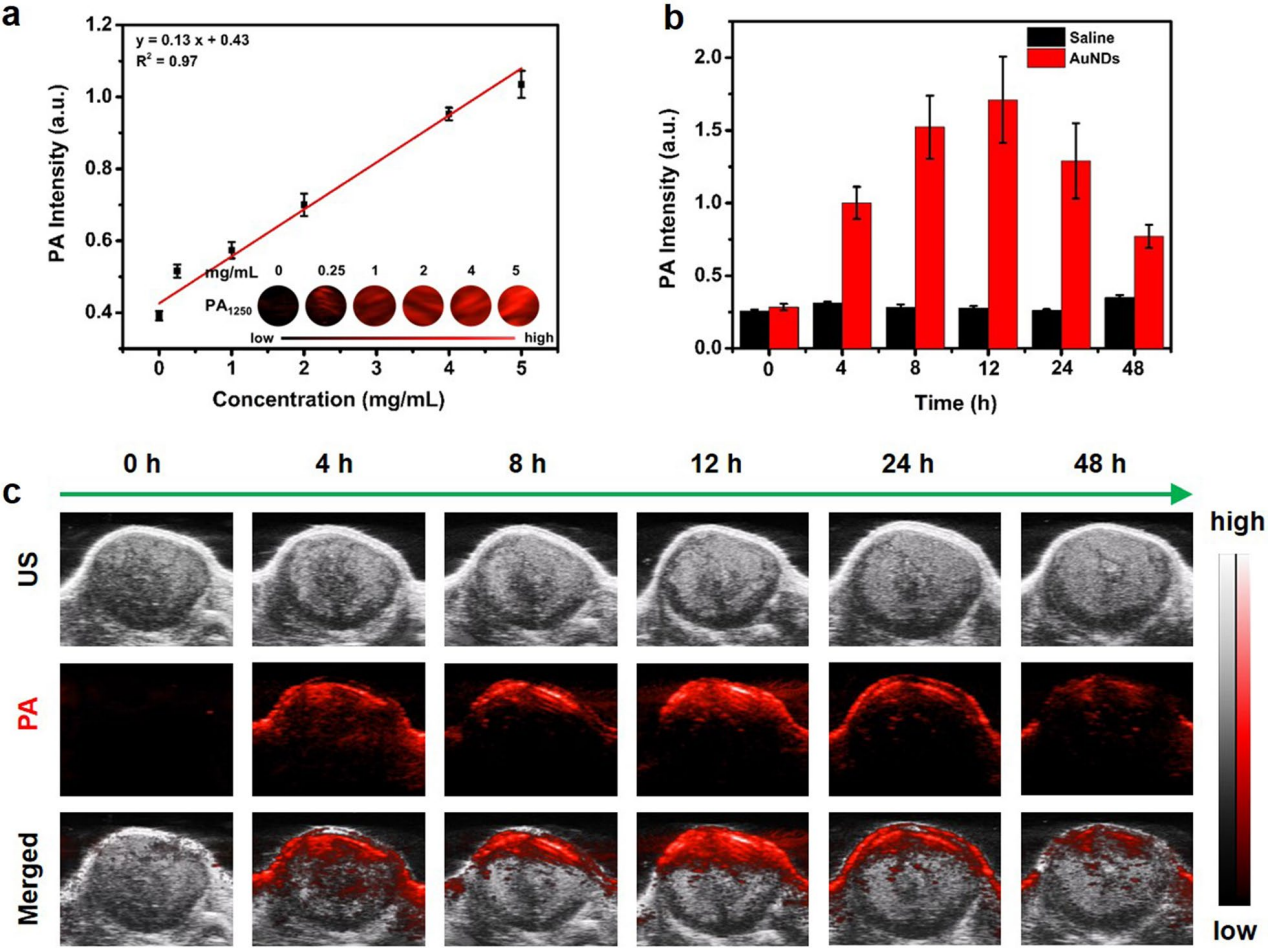
In vitro and in vivo PA imaging. **a** In vitro PA signal intensities at 1250 nm and corresponding PA images of AuNDs aqueous solution at different concentrations (0, 0.25, 1.0, 2.0, 4.0, 5.0 mg/mL). **b, c** In vivo PA signal intensities at 1250 nm and corresponding PA images combined with ultrasonic images of tumorous tissue at varied time points (0, 4, 8, 12, 24, 48 h) after intravenous injection of AuNDs aqueous solution

Encouraged by the strong NIR-II absorption and excellent photothermal performance in vitro, the capability of AuNDs as contrast agents for PAI was further explored. As shown in Fig. 5a and Additional file 1: Fig. S15, the intensities of PA signal displayed a dose-dependent increase and a linear relationship between the concentration of AuNDs and PA intensity was acquired. Then in vivo PAI was also assessed in HCT 116 tumor-bearing mice at various time points. As shown in Fig. 5b, c, after intravenous administration of AuNDs, the PA signal intensities at the tumor site increased first and then reached the maximum value at 12 h. However, the control group without AuNDs displayed negligible PA signals during the course of the experiment. These results strongly demonstrated the feasibility of AuNDs as PA contrast agents in vitro and in vivo, which could be utilized for PAI-guided anti-tumor therapy [44].

### In vivo therapeutic effect of PTT

**Fig. 6:**
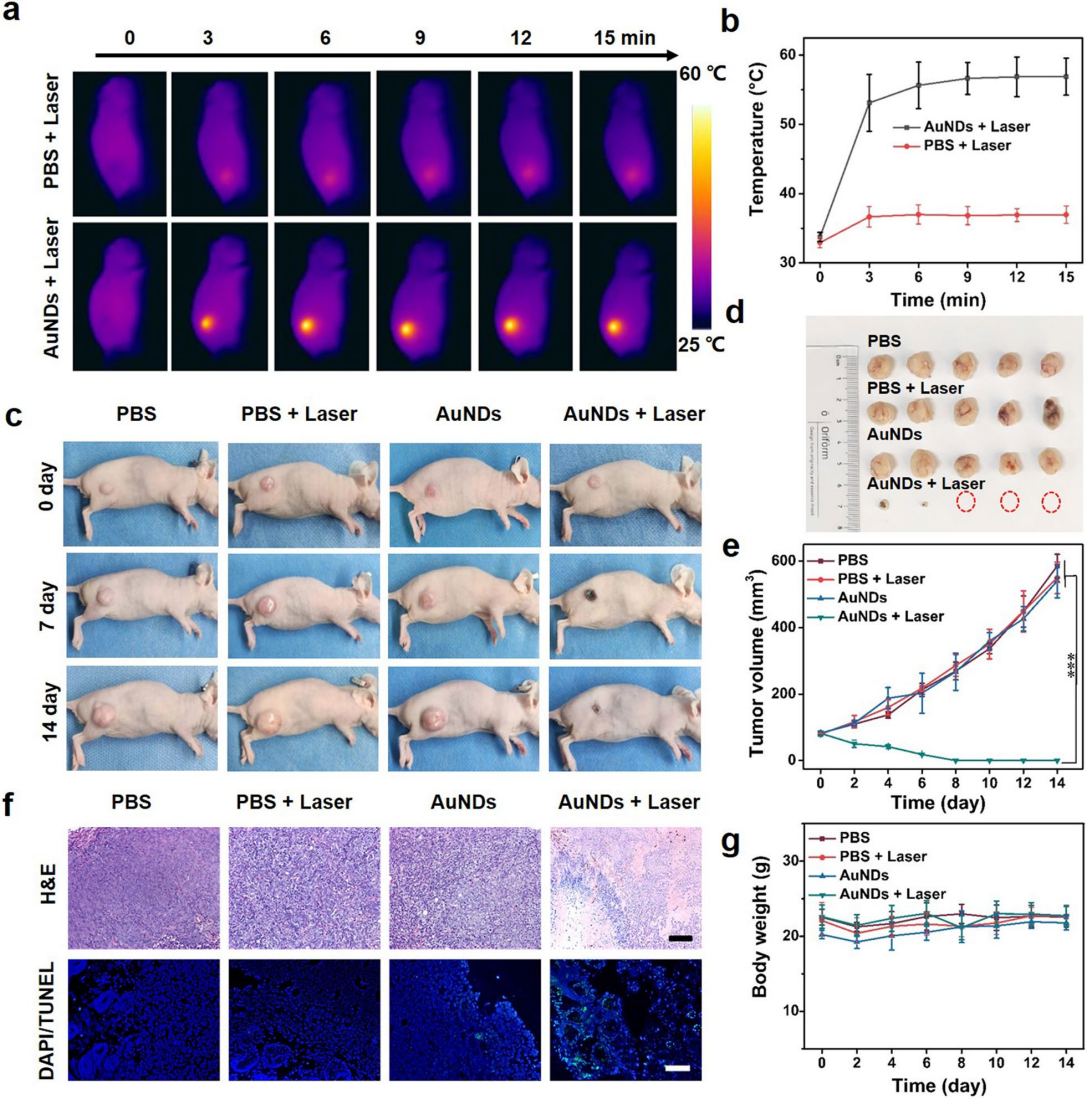
In vivo photothermal and therapeutic effect of AuNDs on HCT 116 tumor-bearing mice. **a** Thermographic images of HCT 116-tumor-bearing mice treated with saline and AuNDs under NIR-II irradiation (1064 nm, 1.0 W cm^−2^). **b** Corresponding photothermal heating curves at the tumor site. **c** Photographs of HCT 116 tumor-bearing mice treated with PBS, PBS + laser, AuNDs, and AuNDs + laser in 14 days. **d** Photograph of excised solid tumor on the 14th day. **e** Tumor volume curves after different treatments [Mean ± SD; n = 5 (students’ t-test, ***P < 0.001)]. **f** H&E and TUNEL stained images of tumor slices on day 14 in different treatment groups (scale bar: 100 μm). **g** Relative body weight changes after different treatments

In an attempt to apply the nanoplatform for in vivo PTT, HCT 116 tumor-bearing mice were exposed to a 1064 nm laser at 12 h post injection of AuNDs. The tumor temperature changes were recorded during the irradiation period. In comparison with PBS-treated mice, the temperature in mice injected with AuNDs increased rapidly from 32 to ~ 52 °C in 15 min under continuous 1064 nm laser irradiation (Fig. 6a, b). In order to study the sustained therapeutic effect of the Au NDs, we monitored tumor volume and body weight every other day during 14-day treatment. The tumors were notably inhibited by AuNDs with laser irradiation, while the other groups showed fast tumor growth with minimal tumor suppression (Fig. 6c–e). The mechanism of tumor destruction was further verified via hematoxylin and eosin (H&E) and terminal deoxynucleotidyl transferase deoxyuridine triphosphate (dUTP) nick end labeling (TUNEL) staining. As expected, serious tumor structure damage by H&E staining and considerable cell apoptosis were observed in Au NDs + laser group, as compared to other groups (Fig. 6f) [45, 46]. In addition, the minimal loss of body weight among all treated mice groups and histopathology tissue analysis presented revealed the negligible systemic toxicity of AuNDs on mice (Fig. 6g and Additional file 1: Fig. S16) [47, 48]. To further assess the long-term biosafety of AuNDs, hemolysis experiment, blood routine and serum biochemistry assay were carried out. The results indicated that AuNDs induced negligible hemolysis, even at high concentration of 1 mg/mL, demonstrating desired biocompatibility of AuNDs (Additional file 1: Fig. S17) [49]. Meanwhile, blood routine and serum biochemistry assays depicted no noticeable variation in the key indicators (Additional file 1: Fig. S18, S19) [50, 51]. Overall, these results collectively indicated that AuNDs had the prominent capability of tumor ablation in vivo with negligible long-term toxicities.

## Conclusions

In summary, we developed a simple CDs-mediated growth strategy to prepare AuNDs with NIR-II absorption for PTT. The AuNDs exhibited two absorption peaks at 1077 and 1265 nm, which could be tuned by the reduction time and concentration of AA. The presence of CDs could promote the formation of branches of AuNDs. Due to the high absorption ability, AuNDs showed good photothermal conversion efficiency of 38% at 1064 nm. The results of theoretical calculation verified that the multi-branch at the edge of AuNDs played an important role in enhancing the localized plasmon heating. Thanks to the good photothermal performance and biocompatibility of AuNDs, the tumor cells could be effectively eliminated in vivo under 1064 nm laser irradiation by the guidance of PAI. Our research not only provides a new method to prepare AuNDs, but also promotes the research of AuNDs in NIR-II window for in vivo PTT.

## Supplementary Information

**Additional file 1: Fig. S1** FT-IR spectrum of CDs. **Fig. S2** a XPS and b N 1s spectra of CDs. **Fig. S3** Energy-dispersive spectroscopy (EDS) spectrum of AuNDs. **Fig. S4** UV–vis–NIR absorption spectra of AuNDs prepared by different concentrations of CDs (controlled by the concentration of AP). **Fig. S5** UV–vis–NIR absorption spectra of AuNDs versus different concentrations of HAuCl4. **Fig. S6** UV–vis–NIR absorption spectra of AuNDs against different concentrations of HCl. **Fig. S7** UV–vis–NIR absorption spectra of AuNDs against various concentrations of PEG-SH. **Fig. S8** TEM images of AuNDs prepared by various concentrations of CDs (controlled by the concentration of AP). **Fig. S9** TEM images of AuNDs prepared by various concentrations of AA. **Fig. S10** Absorption spectra of AuNDs prepared by various concentrations of AA.**Fig. S11** Absorption intensities at 1064 nm versus different concentrations of AuNDs. **Fig. S12** UV-vis-NIR absorbance spectra of AuNDs aqueous solution before and after 1064 nm laser irradiation (1 W cm^-2^) for 10 min. **Table S1** Comparison of photothermal conversion efficiency at 1064 nm of different nanomaterials. **Fig. S14** DLS measurements of AuNDs dispersed in various physiological solutions at different time. **Fig. S15** In vitro PA signal intensities at 1064 nm and corresponding PA images of AuNDs aqueous solution at different concentrations (0, 0.01, 0.05, 0.1, 0.2, 0.3, 0.5 mg/mL). **Fig. S16** H&E-stained tissue section images of PBS (control) and AuNDs treated-healthy mice (scale bar: 50 μm). **Fig. S17** Hemolysis test of AuNDs at different concentrations (12.5, 50, 200, 600 and 1000 μg/mL), and deionized water and PBS as the positive (+) and negative (−) controls, respectively. **Fig. S18** Blood routine analysis of PBS (control) and AuNDs treated-healthy mice. The index analysis included lymphocyte (Lym), hemoglobin (HGB), red blood cell count (RBC), mean platelet volume (MPV), white blood cell count (WBC), and mean corpuscular volume (MCV). **Fig. S19** Serum biochemistry assays of PBS (control) and AuNDs treated-healthy mice. The index analysis included total protein (TP), urea (UREA), uric acid (UA), aspartate aminotransferase (AST), alanine aminotransferase (ALT), and albumin (ALB). **Fig. S13** **a** Schematic illustration of detecting tissue penetration capability of different lasers. **b** Temperature changes of AuNDs aqueous solution versus different thicknesses of chicken breast tissues under 1064 or 808 nm lasers (1 W cm^−2^).

## Data Availability

The data that support the findings of this study are available from the corresponding authors upon reasonable.

## Abbreviations

PTT: Photothermal therapy
NIR-I: First near-infrared window
NIR-II: Second near-infrared window
MPE: Maximum permissible exposure
PAI: Photoacoustic imaging
LSPR: Localized surface plasmon resonance
CDs: Carbon dots
MOs: Metal oxides
AuNDs: Gold nanodendrites
PEG-SH: Poly(ethylene glycol)methyl ether thiol
HCT 116: Human colon cancer cells
PBS: Phosphate buffered saline
EDA: Ethylenediamine
AP: 4-Aminophenol
CCK8: Cell counting kit 8
Calcein AM/PI: Calcein acetoxymethyl ester/propidium iodide
PVDF: Polyvinylidene fluoride
DMEM: Dulbecco’s modified Eagle’s medium
FBS: Fetal bovine serum
HSP: Heat shock protein
HRP: Horseradish peroxidase
BSA: Bovine serum albumin
TEM: Transmission electron microscopy
XPS: X-ray photoelectron spectroscopy
FT-IR: Fourier transform infrared spectroscopy
DLS: Dynamic light scattering
ICP-MS: Inductively coupled plasma mass spectrometry
FDTD: Finite-difference time-domain
H&E: Hematoxylin and eosin
TUNEL: Terminal deoxynucleotidyl transferase deoxyuridine triphosphate (dUTP) nick end labeling

## References

[1] Tan B, Zhao C, Wang J, Tiemuer A, Zhang Y, Yu H, Liu Y (2023). Rational design of pH-activated upconversion luminescent nanoprobes for bioimaging of tumor acidic microenvironment and the enhancement of photothermal therapy. Acta Biomater.

[2] Zhao L, Zhang X, Wang X, Guan X, Zhang W, Ma J (2021). Recent advances in selective photothermal therapy of tumor. J Nanobiotechnol.

[3] Xu C, Pu K (2021). Second near-infrared photothermal materials for combinational nanotheranostics. Chem Soc Rev.

[4] Zhang J, Ning L, Zeng Z, Pu K (2021). Development of second near-infrared photoacoustic imaging agents. Trends Chem.

[5] Yu Z, Chan WK, Zhang Y, Tan TTY (2021). Near-infrared-II activated inorganic photothermal nanomedicines. Biomaterials.

[6] Fu Q, Zhang X, Song J, Yang H (2021). Plasmonic gold nanoagents for cancer imaging and therapy. View.

[7] Sztandera K, Gorzkiewicz M, Klajnert-Maculewicz B (2019). Gold nanoparticles in cancer treatment. Mol Pharm.

[8] Yang X, Yang M, Pang B, Vara M, Xia Y (2015). Gold nanomaterials at work in biomedicine. Chem Rev.

[9] Zhong Q, Feng J, Jiang B, Fan Y, Zhang Q, Chen J, Yin Y (2021). Strain-modulated seeded growth of highly branched black Au superparticles for efficient photothermal conversion. J Am Chem Soc.

[10] Chen J, Gong M, Fan Y, Feng J, Han L, Xin HL, Cao M, Zhang Q, Zhang D, Lei D, Yin Y (2022). Collective plasmon coupling in gold nanoparticle clusters for highly efficient photothermal therapy. ACS Nano.

[11] Lv Z, He S, Wang Y, Zhu X (2021). Noble metal nanomaterials for NIR-triggered photothermal therapy in cancer. Adv Healthcare Mater.

[12] Kuthala N, Shanmugam M, Kong X, Chiang CS, Hwang KC (2022). Salt-mediated, plasmonic field-field/field-lattice coupling-enhanced NIR-II photodynamic therapy using core-gap-shell gold nanopeanuts. Nanoscale Horiz.

[13] Tsai MF, Chang SHG, Cheng FY, Shanmugam V, Cheng YS, Su CH, Yeh CS (2013). Au nanorod design as light-absorber in the first and second biological near-infrared windows for in vivo photothermal therapy. ACS Nano.

[14] Xiong J, Bian Q, Lei S, Deng Y, Zhao K, Sun S, Fu Q, Xiao Y, Cheng B (2021). Bi_19_S_27_I_3_ nanorods: a new candidate for photothermal therapy in the first and second biological near-infrared windows. Nanoscale.

[15] Chen J, Chen T, Fang Q, Pan C, Akakuru OU, Ren W, Lin J, Sheng A, Ma X, Wu A (2022). Gd_2_O_3_/b-TiO_2_ composite nanoprobes with ultra-high photoconversion efficiency for MR image-guided NIR-II photothermal therapy. Exploration.

[16] Li J, Gong X (2022). The emerging development of multicolor carbon dots. Small.

[17] Lu S, Yang B (2022). Carbon dots are shining in the world. SmartMat.

[18] Yan Y, Gong J, Chen J, Zeng Z, Huang W, Pu K, Liu J, Chen P (2019). Recent advances on graphene quantum dots: from chemistry and physics to applications. Adv Mater.

[19] Xia C, Zhu S, Feng T, Yang M, Yang B (2019). Evolution and synthesis of carbon dots: from carbon dots to carbonized polymer dots. Adv Sci.

[20] Liu H, Liu Z, Wang Y, Zhang J, Yang Z, Hu H, Zhao Q, Ning H, Zhi L, Wu M (2021). Carbon dots-oriented synthesis of fungus-like CoP microspheres as a bifunctional electrocatalyst for efficient overall water splitting. Carbon.

[21] Zhang Y, Foster CW, Banks CE, Shao L, Hou H, Zou G, Chen J, Huang Z, Ji X (2016). Graphene-rich wrapped petal-like rutile TiO_2_ tuned by carbon dots for high-performance sodium storage. Adv Mater.

[22] Ye Y, Wang H, Liu H, Xiang Y, Liu L, Deng W, Zou G, Liu Y, Hou H, Ji X (2022). Carbon dots-regulated pomegranate-like metal oxide composites: from growth mechanism to lithium storage. Small Methods.

[23] Ou C, Na W, Ge W, Huang H, Gao F, Zhong L, Zhao Y, Dong X (2021). Biodegradable charge-transfer complexes for glutathione depletion induced ferroptosis and NIR-II photoacoustic imaging guided cancer photothermal therapy. Angew Chem Int Ed.

[24] Liu G, Zhao J, Lu S, Wang S, Sun J, Yang X (2018). Polymethyldopa nanoparticles-based fluorescent sensor for detection of tyrosinase activity. ACS Sens.

[25] Jing Y, Liu G, Zhang C, Yu B, Sun J, Lin D, Qu J (2022). Lipophilic red-emitting carbon dots for detecting and tracking lipid droplets in live cells. ACS Appl Bio Mater.

[26] Ding H, Yu SB, Wei JS, Xiong HM (2016). Full-color light-emitting carbon dots with a surface-state-controlled luminescence mechanism. ACS Nano.

[27] Han L, Liu SG, Dong JX, Liang JY, Li LJ, Li NB, Luo HQ (2017). Facile synthesis of multicolor photoluminescent polymer carbon dots with surface-state energy gap-controlled emission. J Mater Chem C.

[28] Wang J, Sun J, Wang Y, Chou T, Zhang Q, Zhang B, Ren L, Wang H (2020). Gold nanoframeworks with mesopores for raman-photoacoustic imaging and photo-chemo tumor therapy in the second near-infrared biowindow. Adv Funct Mater.

[29] Sun J, Wang J, Hu W, Wang Y, Chou T, Zhang Q, Zhang B, Yu Z, Yang Y, Ren L, Wang H (2021). Camouflaged gold nanodendrites enable synergistic photodynamic therapy and NIR biowindow II photothermal therapy and multimodal imaging. ACS Appl Mater Interfaces.

[30] Cai Q, Wang C, Gai S, Yang P (2022). Integration of Au nanosheets and GdOF:Yb, Er for NIR-I and NIR-II light-activated synergistic theranostics. ACS Appl Mater Interfaces.

[31] Sun Y, Zhang P, Li Y, Hou Y, Yin C, Wang Z, Liao Z, Fu X, Li M, Fan C (2022). Light-activated gold-selenium core-shell nanocomposites with NIR-II photoacoustic imaging performances for heart-targeted repair. ACS Nano.

[32] Gao B, Haghighatbin MA, Cui H (2020). Polymer-encapsulated cobalt/gold bimetallic nanoclusters as stimuli-responsive chemiluminescent nanoprobes for reactive oxygen species. Anal Chem.

[33] Cai R, Xiang H, Yang D, Lin KT, Wu Y, Zhou R, Gu Z, Yan L, Zhao Y, Tan W (2021). Plasmonic AuPt@CuS heterostructure with enhanced synergistic efficacy for radiophotothermal therapy. J Am Chem Soc.

[34] Kim S, Palani S, Civitci F, Nan X, Ibsen S (2022). A versatile synthetic pathway for producing mesostructured plasmonic nanostructures. Small.

[35] Jia T, Li D, Du J, Fang X, Gerasimov V, Agren H, Chen G (2022). A bimodal type of AgPd plasmonic blackbody nanozyme with boosted catalytic efficacy and synergized photothermal therapy for efficacious tumor treatment in the second biological window. J Nanobiotechnol.

[36] Zhou J, Jiang Y, Hou S, Upputuri PK, Wu D, Li J, Wang P, Zhen X, Pramanik M, Pu K, Duan H (2018). Compact plasmonic blackbody for cancer theranosis in the near-infrared II window. ACS Nano.

[37] Linic S, Aslam U, Boerigter C, Morabito M (2015). Photochemical transformations on plasmonic metal nanoparticles. Nat Mater.

[38] Awiaz G, Lin J, Wu A (2023). Recent advances of Au@Ag core–shell sers-based biosensors. Exploration.

[39] Zheng Z, Jia Z, Qin Y, Dai R, Chen X, Ma Y, Xie X, Zhang R (2021). All-in-one zeolite-carbon-based nanotheranostics with adjustable NIR-II window photoacoustic/fluorescence imaging performance for precise NIR-II photothermal-synergized catalytic antitumor therapy. Small.

[40] Xue C, Li M, Liu C, Li Y, Fei Y, Hu Y, Cai K, Zhao Y, Luo Z (2021). NIR-actuated remote activation of ferroptosis in target tumor cells through a photothermally responsive iron-chelated biopolymer nanoplatform. Angew Chem Int Ed.

[41] Duo Y, Luo G, Li Z, Chen Z, Li X, Jiang Z, Yu B, Huang H, Sun Z, Yu XF (2021). Photothermal and enhanced photocatalytic therapies conduce to synergistic anticancer phototherapy with biodegradable titanium diselenide nanosheets. Small.

[42] Zhu Q, Jiang W, Ye K, Jin S, Dong W, Liu S, Zhang G, Tian C, Luo Y, Wang Y, Jiang J (2022). Hydrogenated oxide material for self-targeting and automatic-degrading photothermal tumor therapy in the NIR-II bio-window. Adv Funct Mater.

[43] Su Y, Wu F, Song Q, Wu M, Mohammadniaei M, Zhang T, Liu B, Wu S, Zhang M, Li A, Shen J (2022). Dual enzyme-mimic nanozyme based on single-atom construction strategy for photothermal-augmented nanocatalytic therapy in the second near-infrared biowindow. Biomaterials.

[44] Zhang Y, Shen Q, Li Q, He P, Li J, Huang F, Wang J, Duan Y, Shen C, Saleem F (2021). Ultrathin two-dimensional plasmonic PtAg nanosheets for broadband phototheranostics in both NIR-I and NIR-II biowindows. Adv Sci.

[45] Zhao S, Zhang L, Deng L, Ouyang J, Xu Q, Gao X, Zeng Z, Liu YN (2021). NIR-II responsive hydrogel as an angiogenesis inhibition agent for tumor microenvironment reprogramming. Small.

[46] Yu H, Ma M, Liang K, Shen J, Lan Z, Chen H (2021). A self-assembled metal-polyphenolic nanomedicine for mild photothermal-potentiated chemodynamic therapy of tumors. Appl Mater Today.

[47] Qian Y, Zhang J, Zou J, Wang X, Meng X, Liu H, Lin Y, Chen Q, Sun L, Lin W, Wang H (2022). NIR-II responsive PEGylated nickel nanoclusters for photothermal enhanced chemodynamic synergistic oncotherapy. Theranostics.

[48] Tiemuer A, Yu H, Zhao C, Sun W, Zhang Y, Jiang Y, Gu Y, Liu Y (2022). Nitroso-caged upconversion luminescent prodrug: near infrared light-activatable NO nano-donor for gas therapy. Chem Eng J.

[49] Sun S, Chen Q, Li Y, Yu Y, Li Z, Lin H (2022). Tumor-specific and photothermal-augmented chemodynamic therapy by ferrocene-carbon dot-crosslinked nanoparticles. SmartMat.

[50] Wang G, Zhang N, Cao Z, Zhang Z, Zhu Z, Sun G, Jin L, Yang X (2021). Injectable hydrogel-mediated combination of hyperthermia ablation and photo-enhanced chemotherapy in the NIR-II window for tumor eradication. Biomater Sci.

[51] Zhou Z, Wang X, Zhang H, Huang H, Sun L, Ma L, Du Y, Pei C, Zhang Q, Li H (2021). Activating layered metal oxide nanomaterials via structural engineering as biodegradable nanoagents for photothermal cancer therapy. Small.

