# Supplementary material for: Carbon dots-mediated synthesis of gold nanodendrites with extended absorption into NIR-II window for in vivo photothermal therapy
Source: J Nanobiotechnology. 2023 May 9;21:151. doi: 10.1186/s12951-023-01887-2 (PMC10170720; doi:10.1186/s12951-023-01887-2)
Supplement: Supplementary file 1 — Additional file 1: Fig. S1 FT-IR spectrum of CDs. Fig. S2 a XPS and b N 1s spectra of CDs. Fig. S3 Energy-dispersive spectroscopy (EDS) spectrum of AuNDs. Fig. S4 UV–vis–NIR absorption spectra of AuNDs prepared by different concentrations of CDs (controlled by the concentration of AP). Fig. S5 UV–vis–NIR absorption spectra of AuNDs versus different concentrations of HAuCl4. Fig. S6 UV–vis–NIR absorption spectra of AuNDs against different concentrations of HCl. Fig. S7 UV–vis–NIR absorption spectra of AuNDs against various concentrations of PEG-SH. Fig. S8 TEM images of AuNDs prepared by various concentrations of CDs (controlled by the concentration of AP). Fig. S9 TEM images of AuNDs prepared by various concentrations of AA. Fig. S10 Absorption spectra of AuNDs prepared by various concentrations of AA.Fig. S11 Absorption intensities at 1064 nm versus different concentrations of AuNDs. Fig. S12 UV-vis-NIR absorbance spectra of AuNDs aqueous solution before and after 1064 nm laser irradiation (1 W cm-2) for 10 min. Table S1 Comparison of photothermal conversion efficiency at 1064 nm of different nanomaterials. Fig. S14 DLS measurements of AuNDs dispersed in various physiological solutions at different time. Fig. S15 In vitro PA signal intensities at 1064 nm and corresponding PA images of AuNDs aqueous solution at different concentrations (0, 0.01, 0.05, 0.1, 0.2, 0.3, 0.5 mg/mL). Fig. S16 H&E-stained tissue section images of PBS (control) and AuNDs treated-healthy mice (scale bar: 50 μm). Fig. S17 Hemolysis test of AuNDs at different concentrations (12.5, 50, 200, 600 and 1000 μg/mL), and deionized water and PBS as the positive (+) and negative (−) controls, respectively. Fig. S18 Blood routine analysis of PBS (control) and AuNDs treated-healthy mice. The index analysis included lymphocyte (Lym), hemoglobin (HGB), red blood cell count (RBC), mean platelet volume (MPV), white blood cell count (WBC), and mean corpuscular volume (MCV). Fig. S19 Serum biochemistry assays [file 12951_2023_1887_MOESM1_ESM.docx]

Supporting Information

Carbon dots-mediated synthesis of gold nanodendrites with extended absorption into NIR-II window for *in vivo* photothermal therapy

Guoyong Liu^1,2†^, Shuxian Wang^3†^, Shumin Wang^1^, Rongrong Wu^4^, Hui Li^1^, Menglei Zha^3^, Jibin Song^4^, Yuxin Yin^1^, Kai Li^3*^, Jing Mu^1*^, and Yu Shi^2*^

^1^Department of Nuclear Medicine, Peking University Shenzhen Hospital, Shenzhen Peking University-The Hong Kong University of Science and Technology Medical Center, Shenzhen 518036, China

^2^Department of Ultrasound, Peking University Shenzhen Hospital, Peking University, Shenzhen, 518036, China

^3^Shenzhen Key Laboratory of Smart Healthcare Engineering, Guangdong Provincial Key Laboratory of Advanced Biomaterials, Department of Biomedical Engineering, Southern University of Science and Technology, Shenzhen 518055, China

^4^MOE Key Laboratory for Analytical Science of Food Safety and Biology, College of Chemistry, Fuzhou University, Fuzhou 350108, China

^†^These authors contributed equally to this work.

*Correspondence: mujing1921@gmail.com; shiyu@pkuszh.com; lik@sustech.edu.cn


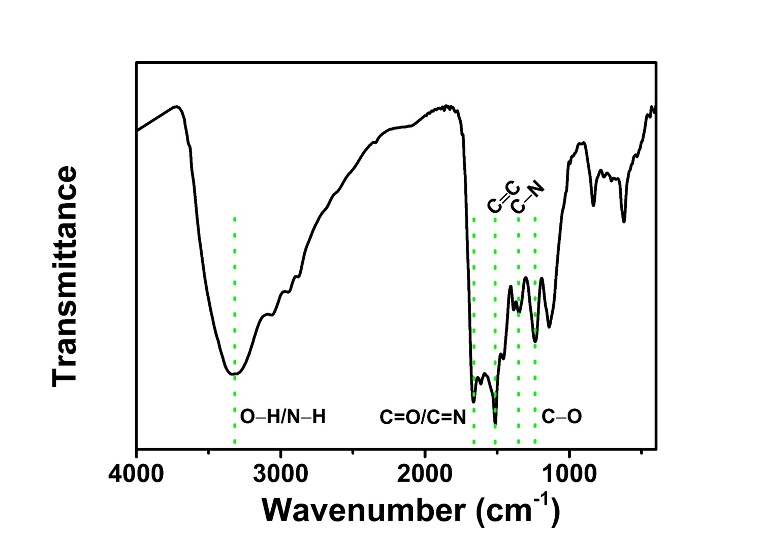


**Fig. S1** FT-IR spectrum of CDs


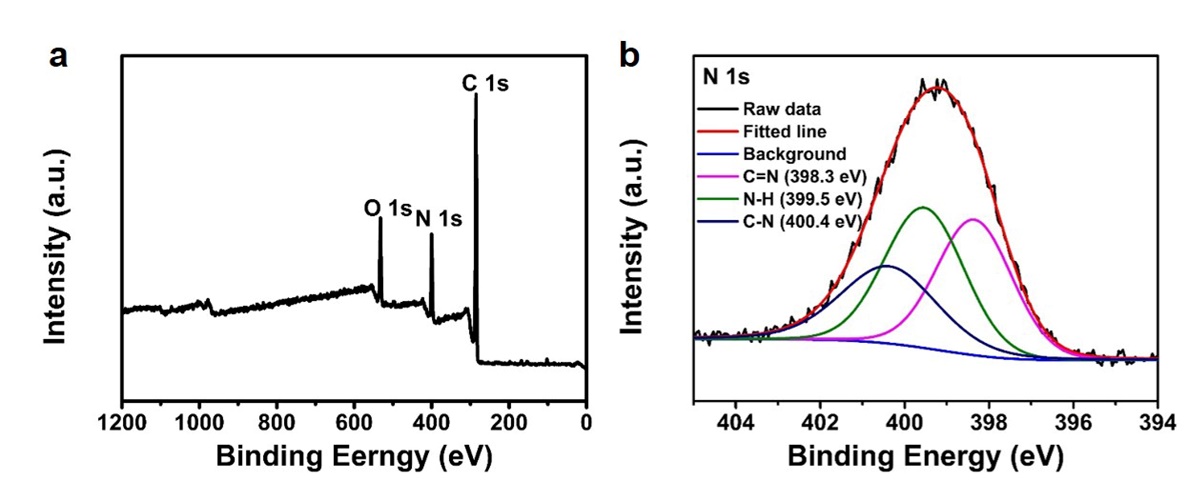


**Fig. S2** **a** XPS and **b** N 1s spectra of CDs.


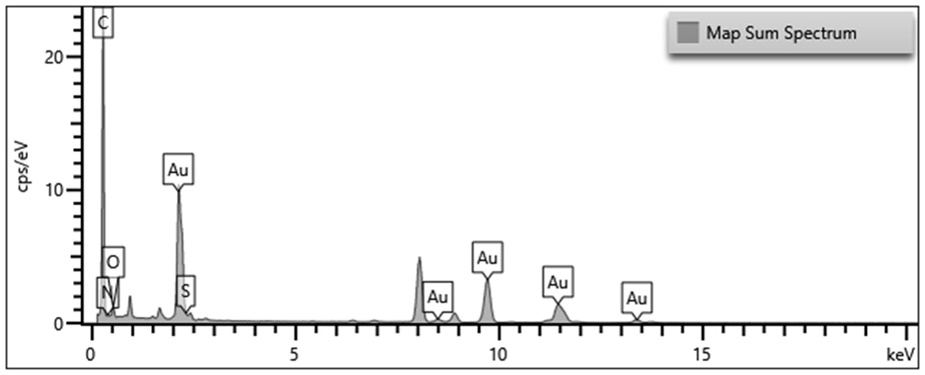


**Fig. S3** Energy-dispersive spectroscopy (EDS) spectrum of AuNDs.


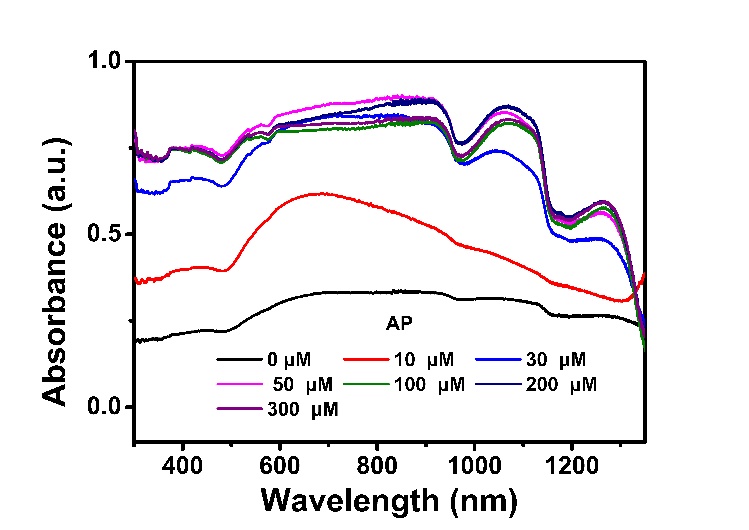


**Fig. S4** UV–vis–NIR absorption spectra of AuNDs prepared by different concentrations of CDs (controlled by the concentration of AP).


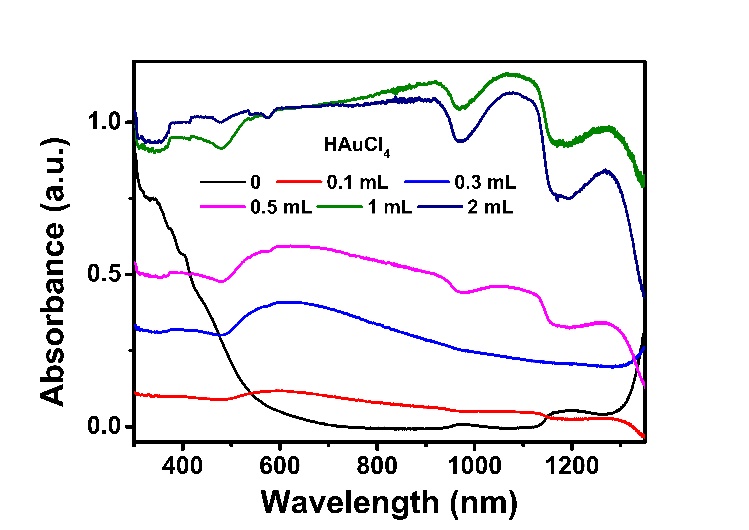


**Fig. S5** UV–vis–NIR absorption spectra of AuNDs versus different concentrations of HAuCl_4_.


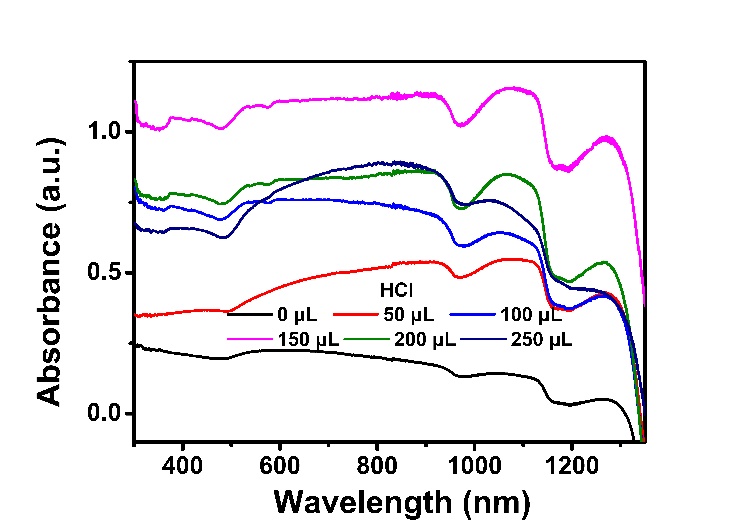


**Fig. S6** UV–vis–NIR absorption spectra of AuNDs against different concentrations of HCl.


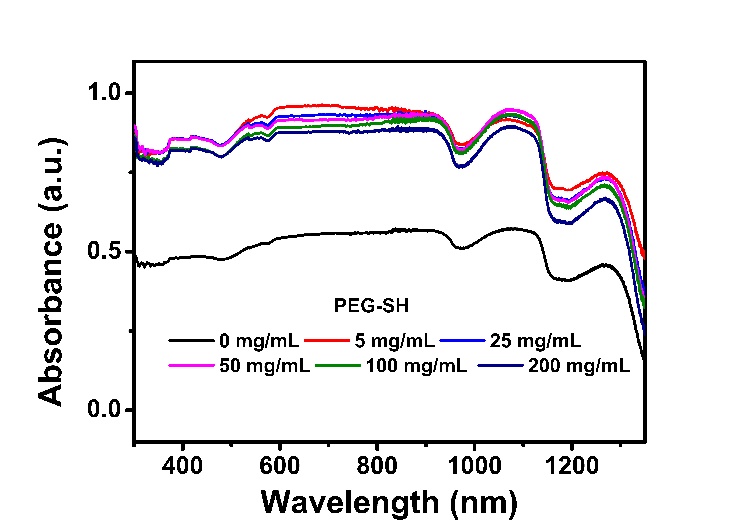


**Fig. S7** UV–vis–NIR absorption spectra of AuNDs against various concentrations of PEG-SH


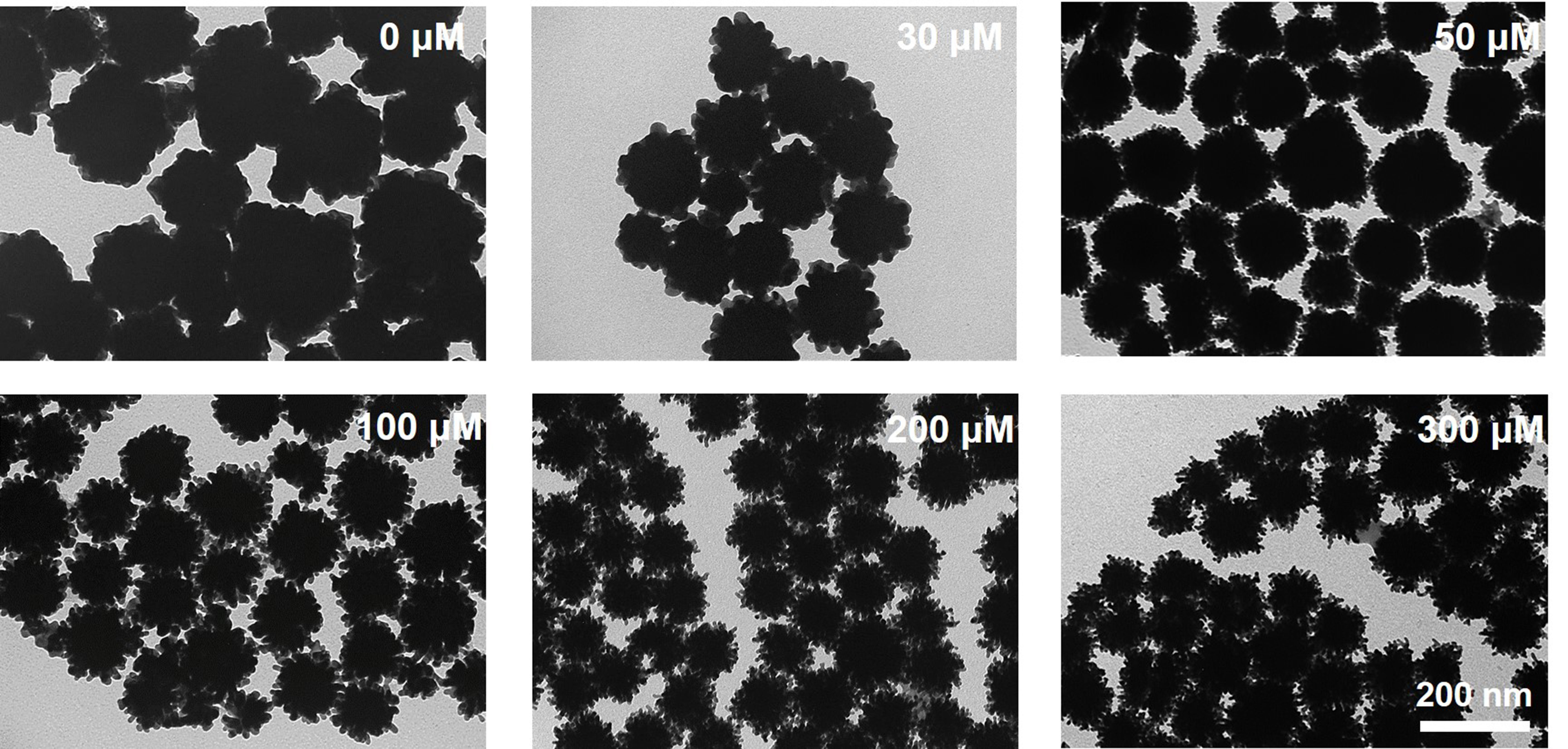


**Fig. S8** TEM images of AuNDs prepared by various concentrations of CDs (controlled by the concentration of AP).


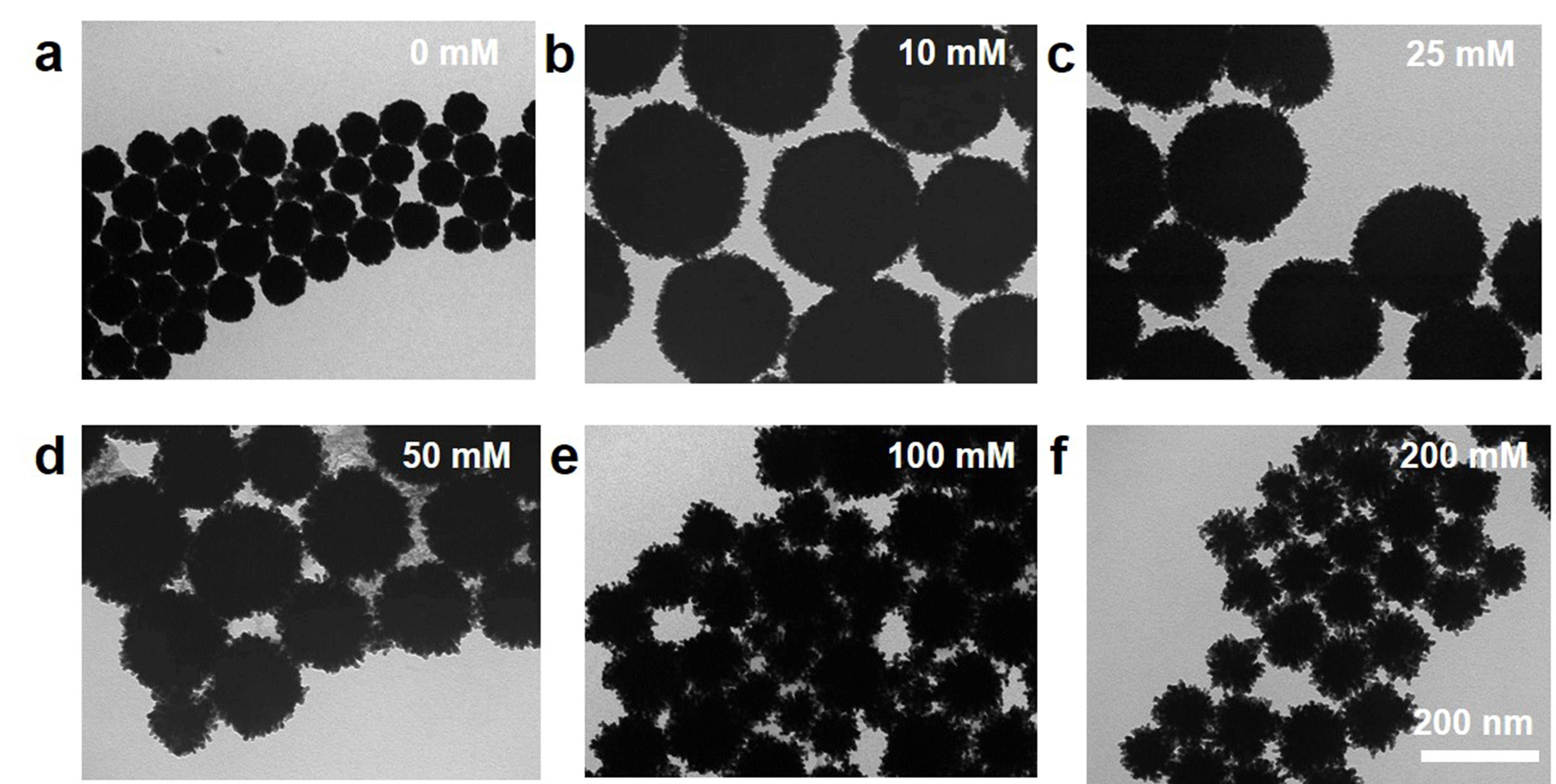


**Fig. S9** TEM images of AuNDs prepared by various concentrations of AA.


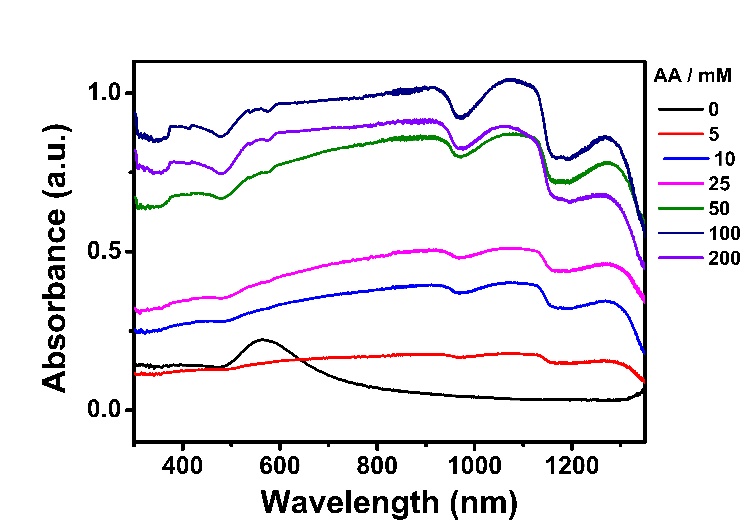


**Fig. S10** Absorption spectra of AuNDs prepared by various concentrations of AA.


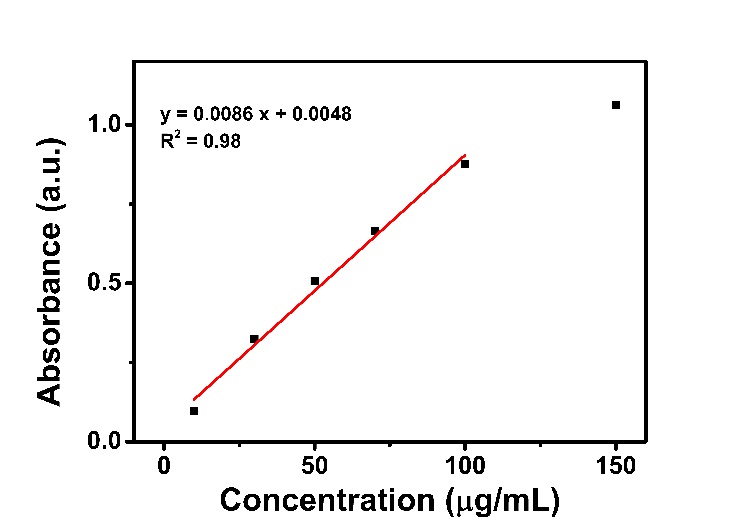


**Fig. S11** Absorption intensities at 1064 nm versus different concentrations of AuNDs.





**Fig S12** UV-vis-NIR absorbance spectra of AuNDs aqueous solution before and after 1064 nm laser irradiation (1 W cm^-2^) for 10 min.

**Table S1** Comparison of photothermal conversion efficiency at 1064 nm of different nanomaterials.

| Materials | Dominant elements | Photothermal conversion efficiency | Reference |
| --- | --- | --- | --- |
| Gold Nanoframeworks | Au | 23.9% | [1] |
| Gold Hollow Nanorods | Au | 33% | [2] |
| Au NPL@TiO_2_ | Au | 42.05% | [3] |
| Bi@C nanoparticles | C | 43.2% | [4] |
| Si–Au NCs | Si | 24.1% | [5] |
| MoO_x_ nanoparticles | Mo | 37.4% | [6] |
| MoO_3_ nanobelts | Mo | 24.27 | [7] |
| Fe_3_O_4_@Ag/Au | Fe | 28.3% | [8] |
| Au−Cu_9_S_5_ | Cu | 37% | [9] |
| Graphene quantum dots | C | 33.45% | [10] |
| AuNDs | Au | 38% | This work |

**Calculation of Photothermal Conversion Efficiency (*η*):** The photothermal conversion efficiency is calculated by formula (1):

$\eta=\frac{hS\left( T_{max}-T_{surr} \right)-Q_{dis}}{I\left( 1-{10}^{-A_{1064}} \right)}$ (1)

where *h* is the heat transfer coefficient, *S* is the surface area of the container, *T_max_* is the maximum temperature of the solution, *T_surr_* is the surrounding temperature, *I* is the laser power, and *A_1064_* is the absorption value of the material at 1064 nm. *Q_dis_* is the heat dissipated from light absorbed by the quartz sample cell containing pure water. To calculate *hS*, equation (2) and (3) were introduced:

$Q_{dis}=hS(T_{max,H_{2}O}-T_{surr})$ (2)

$\tau_{s}=\frac{m_{D}C_{D}}{hS}$ (3)

*m_D_* is the mass of water, *C_D_* is the heat capacity of water (4.2 J·g^-1^·°C^-1^), τ*_s_* is the sample system time constant, which was calculated by formula (4) and (5):

$t=-\tau_{s}ln\theta$ (4)

$\theta=\frac{T_{surr}-T}{T_{surr}{-T}_{max}}$ (5)


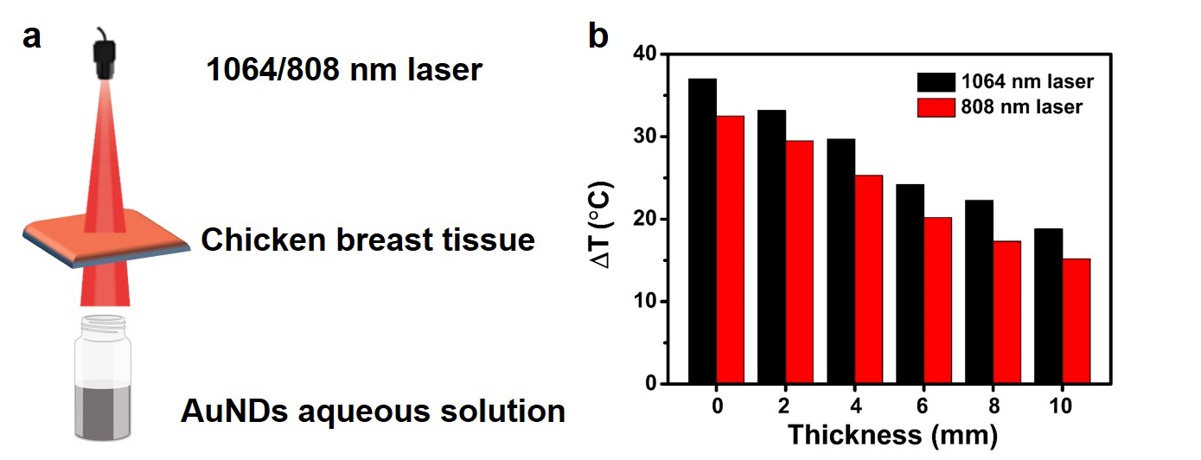


**Fig. S13** **a** Schematic illustration of detecting tissue penetration capability of different lasers. **b** Temperature changes of AuNDs aqueous solution versus different thicknesses of chicken breast tissues under 1064 or 808 nm lasers (1 W cm^−2^).


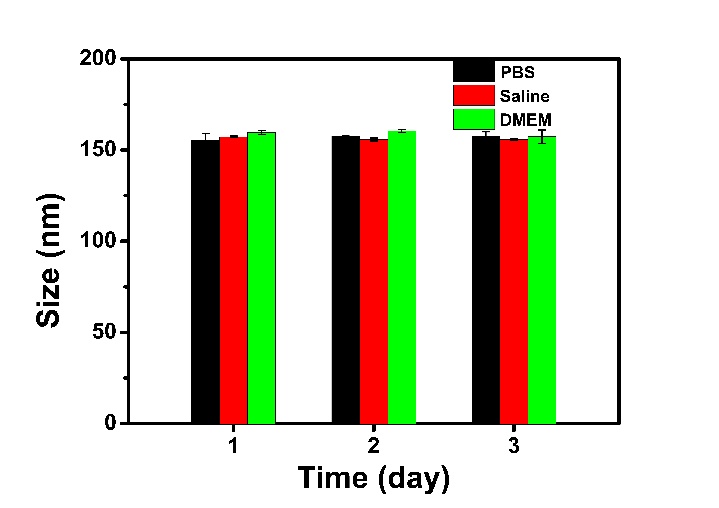


**Fig. S14** DLS measurements of AuNDs dispersed in various physiological solutions at different time.


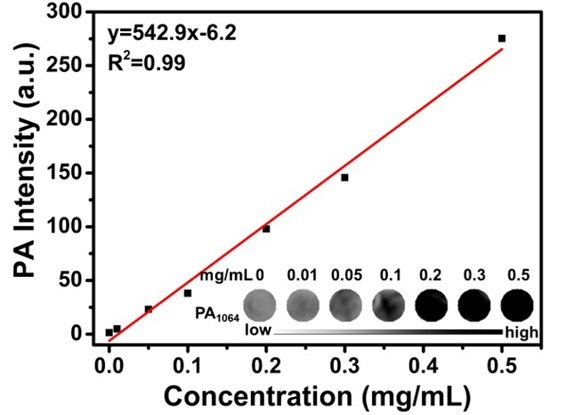


**Fig. S15** *In* *vitro* PA signal intensities at 1064 nm and corresponding PA images of AuNDs aqueous solution at different concentrations (0, 0.01, 0.05, 0.1, 0.2, 0.3, 0.5 mg/mL).


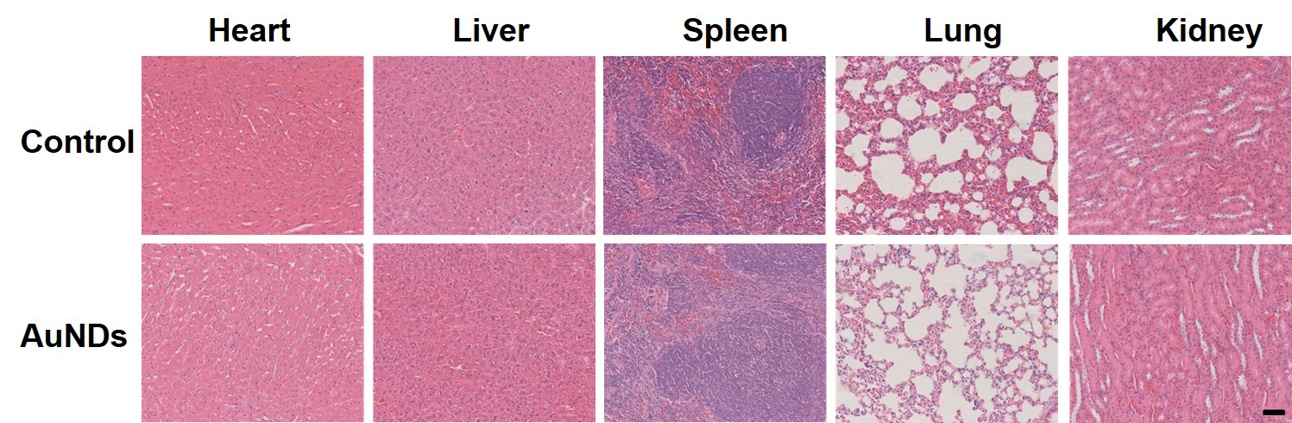


**Fig. S16** H&E-stained tissue section images of PBS (control) and AuNDs treated-healthy mice (scale bar: 50 μm).


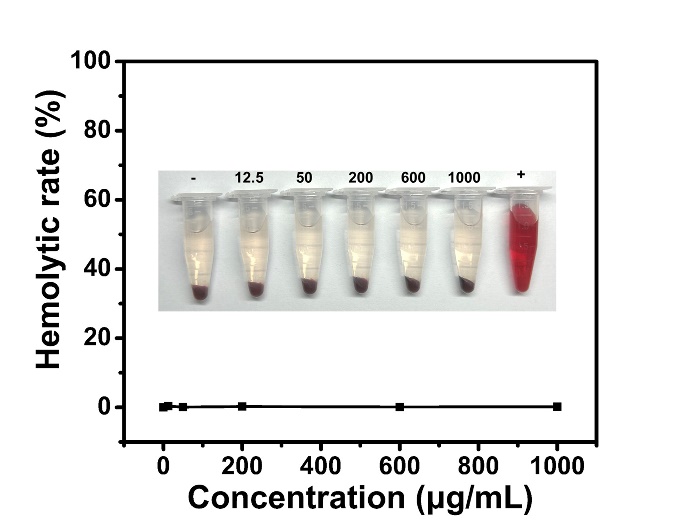


**Fig. S17** Hemolysis test of AuNDs at different concentrations (12.5, 50, 200, 600 and 1000 μg/mL), and deionized water and PBS as the positive (+) and negative (−) controls, respectively.


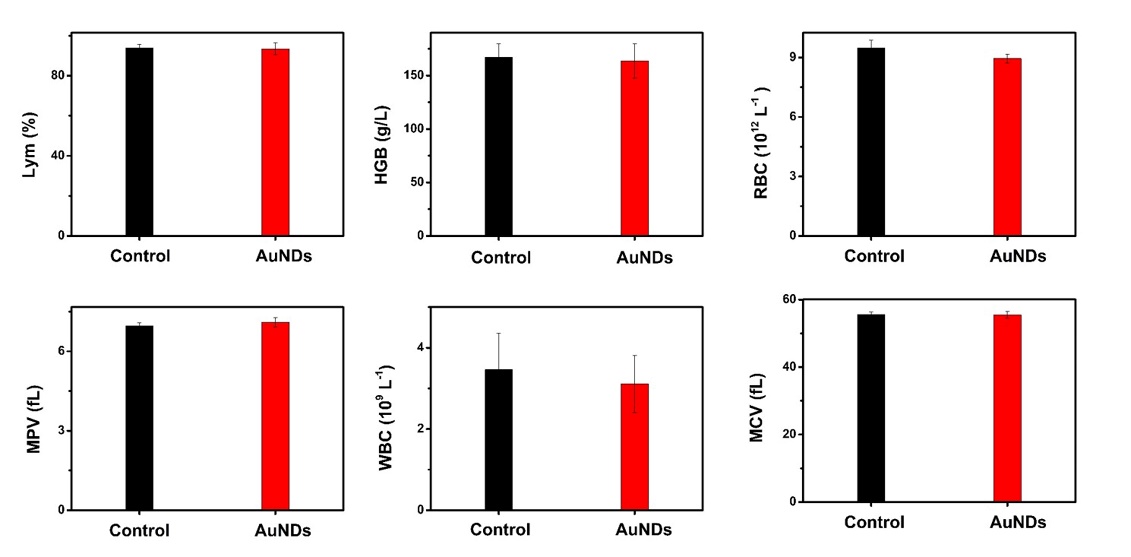


**Fig. S18** Blood routine analysis of PBS (control) and AuNDs treated-healthy mice. The index analysis included lymphocyte (Lym), hemoglobin (HGB), red blood cell count (RBC), mean platelet volume (MPV), white blood cell count (WBC), and mean corpuscular volume (MCV).


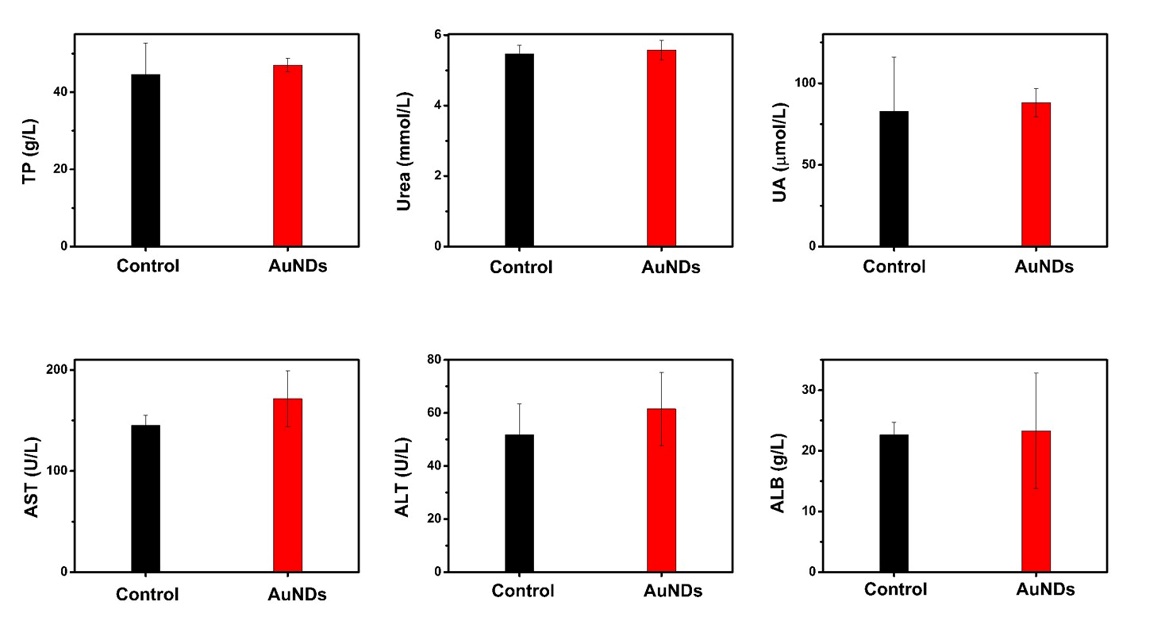


**Fig. S19** Serum biochemistry assays of PBS (control) and AuNDs treated-healthy mice. The index analysis included total protein (TP), urea (UREA), uric acid (UA), aspartate aminotransferase (AST), alanine aminotransferase (ALT), and albumin (ALB).

**References**

1. Wang J, Sun J, Wang Y, Chou T, Zhang Q, Zhang B, Ren L, Wang H. Gold nanoframeworks with mesopores for raman-photoacoustic imaging and photo-chemo tumor therapy in the second near-infrared biowindow. Adv Funct Mater. 2020;30(9):1908825.

2. Cai K, Zhang W, Zhang J, Li H, Han H, Zhai T. Design of gold hollow nanorods with controllable aspect ratio for multimodal imaging and combined chemo-photothermal therapy in the second near-infrared window. ACS Appl Mater Interfaces. 2018;10(43):36703-10.

3. Gao F, He G, Yin H, Chen J, Liu Y, Lan C, Zhang S, Yang B. Titania-coated 2D gold nanoplates as nanoagents for synergistic photothermal/sonodynamic therapy in the second near-infrared window. Nanoscale. 2019;11(5):2374-84.

4. Zhen W, An S, Wang W, Liu Y, Jia X, Wang C, Zhang M, Jiang X. Gram-scale fabrication of Bi@C nanoparticles through one-step hydrothermal method for dual-model imaging-guided NIR-II photothermal therapy. Nanoscale. 2019;11(20):9906-11.

5. Sun L, Chen Y, Gong F, Dang Q, Xiang G, Cheng L, Liao F, Shao M. Silicon nanowires decorated with gold nanoparticles via *in situ* reduction for photoacoustic imaging-guided photothermal cancer therapy. J Mater Chem B. 2019;7(28):4393-401.

6. Yin W, Bao T, Zhang X, Gao Q, Yu J, Dong X, Yan L, Gu Z, Zhao Y. Biodegradable MoO_x_ nanoparticles with efficient near-infrared photothermal and photodynamic synergetic cancer therapy at the second biological window. Nanoscale. 2018;10(3):1517-31.

7. Zhou Z, Wang Y, Peng F, Meng F, Zha J, Ma L, Du Y, Peng N, Ma L, Zhang Q, et al. Intercalation-activated layered MoO_3_ nanobelts as biodegradable nanozymes for tumor-specific photo-enhanced catalytic therapy. Angew Chem Int Ed. 2022;61(16):202115939.

8. Tsai MF, Hsu C, Yeh CS, Hsiao YJ, Su CH, Wang LF. Tuning the distance of rattle-shaped ionp@shell-in-shell nanoparticles for magnetically-targeted photothermal therapy in the second near-infrared window. ACS Appl Mater Interfaces. 2018;10(2):1508-19.

9. Ding X, Liow CH, Zhang M, Huang R, Li C, Shen H, Liu M, Zou Y, Gao N, Zhang Z, et al. Surface plasmon resonance enhanced light absorption and photothermal therapy in the second near-infrared window. J Am Chem Soc. 2014;136(44):15684-93.

10. Liu H, Li C, Qian Y, Hu L, Fang J, Tong W, Nie R, Chen Q, Wang H. Magnetic-induced graphene quantum dots for imaging-guided photothermal therapy in the second near-infrared window. Biomaterials. 2020;232:119700.
